# Supplementary material for: Perspectives on simulation-based training from paediatric healthcare providers in Nigeria: a national survey
Source: BMJ Open. 2020 Feb 10;10(2):e034029. doi: 10.1136/bmjopen-2019-034029 (PMC7044915; doi:10.1136/bmjopen-2019-034029)
Supplement: Supplementary data [file bmjopen-2019-034029supp001.pdf]

Survey on simulation based learning/practice

Please answer the following questions:

1. **What is your age range?**
   a. <21 **b.** 21-30 **c.** 31-40 **d.** 41-50 **e.** >50
2. **Sex**
   a. Male b. Female
3. **Profession**
   a. Consultant physician **b.** Senior resident/registrar **c.** Resident/registrar **d.** Nurse **e.** Other _________
4. **Years of practice**
   a. < 5 years **b.** 5-10 years **c.** 11-15 years **d.** 16-20 years **e.** >20 years
5. **Current location of practice**
   ____
6. **Type of practice**
   a. Government facility
      i. Tertiary care
      ii. Secondary care
      iii. Primary care
   b. Private practice
   c. Faith-based facility
7. **Specialty**
   a. General Pediatrics
   b. Subspecialty Pediatrics (Please specify ___________________)
   c. Other specialty (Please specify ___________________)
8. **Are you aware of the use of simulation-based training at your institution?**
   a. Yes **b.** No
9. **If yes in Q8, in what capacity does your institution use simulation-based training? (select all that apply)**
   a. Teaching **b.** Research **c.** Examination
10. **Does your center have a skills-based simulation lab?**
    a. Yes **b**. No
11. **If yes in Q10, what is the skills-based simulation lab available for? (select all that apply)**
    a. Skills practice e.g. HBB Newborn corner **b.** Teaching **c.** Research **d.** Examination
12. **If no in Q10, what are the challenges to having a skills-based simulation lab at your center?**
    a. Lack of funding

- **b.** Lack of access to equipment e.g. manikins
- **c.** Lack of curriculum
- **d.** Lack of space
- **e.** Lack of instructors trained in simulation education
- **f.** Lack of awareness of an option for simulation-based training

13. Which modality of simulation based training have you been exposed to? (select all that apply)

- **a.** Manikin-based training
  - i. HBB
  - ii. NRT
  - iii. PALS
  - iv. ENCC
  - v. BLS
- **b.** Online (computer-based) simulation
  - i. NRP eSIM™
  - ii. HeartCode™ (PALS online course)
  - iii. Online Basic Life Support course
  - iv. Online ACLS course

14. Are you aware of virtual reality simulation training?

- **a.** Yes **b.** No

15. If yes in Q14, when or where were you exposed to virtual reality simulation?

____

16. If no in Q14, what are the challenges to online (computer-based or virtual reality) simulation?

- **a.** Lack of internet access
- **b.** Lack of standardized VR training modules
- **c.** Inconsistent power supply
- **d.** Lack of access to VR equipment and computers

17. Which of these advantages of simulation-based training are you aware of (select all that apply)?

- **a.** Skills acquisition
- **b.** Provides feedback
- **c.** Step down training
- **d.** Monitoring and evaluation
- **e.** Debriefing/reflection
- **f.** Hands-on skills practice
- **g.** Teamwork/communication training
- **h.** Skills maintenance/retention
- **i.** Examination purposes when patients are unavailable

18. What type of mobile phone device do you own or use? (Choose all that apply)

- **a.** Tablet (Eg. Ipad, tablets)

Umoren R, *et al. BMJ Open* 2020; 10:e034029. doi: 10.1136/bmjopen-2019-034029

- **b.** Smart Phone (eg. Iphone, Samsung, Techno, Nexus, Infinix etc.)
- **c.** Feature phone (Eg. Does some gprs based activities)
- **d.** Basic (Eg. Used for call and SMS only)

19. What is the manufacturer and model of your phone/mobile device?

____

20. If you are using an android enabled device, what android version does your device run (To find out, Goto |Settings->General->AboutDevice| and look for version number)

- **a.** Gingerbread (version 2.3)
- **b.** Ice Cream Sandwich (version 4.0)
- **c.** Jelly Bean (version 4.1 – 4.3)
- **d.** KitKat (Version 4.4)
- **e.** Lollipop (Version 5.0 – 5.1)
- **f.** Marshmallow (Version 6.0)
- **g.** Nougat (Version 7)
- **h.** Oreo (Version 8)

21. Do you use mobile device currently for your work?

a. Yes **b.** No

22. Do you think simulation based training could be expanded beyond the current scope?

a. Yes **b.** No

23. If yes in Q22, in what way should simulation-based training be expanded in Nigeria?

- **a.** Continued practice after initial training
- **b.** Teaching
- **c.** Research

24. If all facilities were available, would you recommend online simulation for your center?

a. Yes b. No

25. If No in Q24, please state your reason(s)____
