# Supplementary material for: Perspectives on simulation-based training from paediatric healthcare providers in Nigeria: a national survey
Source: BMJ Open. 2020 Feb 10;10(2):e034029. doi: 10.1136/bmjopen-2019-034029 (PMC7044915; doi:10.1136/bmjopen-2019-034029)
Supplement: Author's manuscript [file bmjopen-2019-034029.draft_revisions.pdf]

BMJ Open is committed to open peer review. As part of this commitment we make the peer review history of every article we publish publicly available.

When an article is published we post the peer reviewers' comments and the authors' responses online. We also post the versions of the paper that were used during peer review. These are the versions that the peer review comments apply to.

The versions of the paper that follow are the versions that were submitted during the peer review process. They are not the versions of record or the final published versions. They should not be cited or distributed as the published version of this manuscript.

BMJ Open is an open access journal and the full, final, typeset and author-corrected version of record of the manuscript is available on our site with no access controls, subscription charges or pay-per-view fees (http://bmjopen.bmj.com).

If you have any questions on BMJ Open's open peer review process please email info.bmjopen@bmj.com

BMJ Open

Perspectives on Simulation-based Education from Paediatric Healthcare Providers in Nigeria: A National Survey

Journal:	*BMJ Open*
Manuscript ID	bmjopen-2019-034029
Article Type:	Original research
Date Submitted by the Author:	03-Sep-2019
Complete List of Authors:	Umoren, Rachel; University of Washington School of Medicine; Seattle Children's Hospital<br>Ezeaka, V; University of Lagos College of Medicine, Paediatrics<br>Fajolu, Ireti; University of Lagos College of Medicine, Paediatrics<br>Ezenwa, Beatrice; University of Lagos College of Medicine, Paediatrics<br>Akintan, Patricia; University of Lagos College of Medicine, Paediatrics<br>Chukwu, Emeka; University of Washington School of Medicine<br>Spiekerman, Chuck; University of Washington School of Medicine
Keywords:	World Wide Web technology < BIOTECHNOLOGY & BIOINFORMATICS, EDUCATION & TRAINING (see Medical Education & Training), MEDICAL EDUCATION & TRAINING, PAEDIATRICS

SCHOLARONE™ Manuscripts

I, the Submitting Author has the right to grant and does grant on behalf of all authors of the Work (as defined in the below author licence), an exclusive licence and/or a non-exclusive licence for contributions from authors who are: i) UK Crown employees; ii) where BMJ has agreed a CC-BY licence shall apply, and/or iii) in accordance with the terms applicable for US Federal Government officers or employees acting as part of their official duties; on a worldwide, perpetual, irrevocable, royalty-free basis to BMJ Publishing Group Ltd ("BMJ") its licensees and where the relevant Journal is co-owned by BMJ to the co-owners of the Journal, to publish the Work in this journal and any other BMJ products and to exploit all rights, as set out in our licence.

The Submitting Author accepts and understands that any supply made under these terms is made by BMJ to the Submitting Author unless you are acting as an employee on behalf of your employer or a postgraduate student of an affiliated institution which is paying any applicable article publishing charge ("APC") for Open Access articles. Where the Submitting Author wishes to make the Work available on an Open Access basis (and intends to pay the relevant APC), the terms of reuse of such Open Access shall be governed by a Creative Commons licence – details of these licences and which Creative Commons licence will apply to this Work are set out in our licence referred to above.

Other than as permitted in any relevant BMJ Author's Self Archiving Policies, I confirm this Work has not been accepted for publication elsewhere, is not being considered for publication elsewhere and does not duplicate material already published. I confirm all authors consent to publication of this Work and authorise the granting of this licence.

For peer review only

Perspectives on Simulation-based Education from Paediatric Healthcare Providers in Nigeria: A National Survey

[1]Umoren RA, [3]Ezeaka VC, [3]Fajolu IB, [3]Ezenwa BN, [3]Akintan P, [1]Chukwu E, [1]Spiekerman, C.
[1]University of Washington, Seattle, Washington, U.S.A.; [2]Seattle Children's Hospital, Seattle, Washington, U.S.A.; [3]College of Medicine, University of Lagos, Lagos, Nigeria

Corresponding Author: Rachel A. Umoren MB.BCh, MS, FAAP. Associate Professor of Pediatrics, Department of Pediatrics, Seattle Children's Hospital & University of Washington, 1959 NE Pacific Street, Box 356320, Seattle, WA 98195. Email: rumoren@uw.edu. Phone: +1-206-543-3200.

Keywords: Education and training, Paediatrics, Medical education and training, World Wide Web technology.

Word count: 2620

For peer review only

ABSTRACT

OBJECTIVES: The objective of this study was to explore the access to and perceived utility of various simulation modalities by in-service healthcare providers in a low resource setting.

SETTING: A paper-based 35-item cross-sectional survey on simulation-based training (SBT) was administered to a convenience sample of healthcare workers from secondary and tertiary healthcare facilities during Paediatric training workshops at a National Paediatric Conference in Nigeria.

PARTICIPANTS: All 200 healthcare workers who attended the workshop sessions were eligible to participate. A total of 161 surveys were completed (RR 81%).

PRIMARY AND SECONDARY OUTCOME MEASURES: The primary outcome measure was an assessment of the perceptions of healthcare providers in a low resource setting towards SBT and their access to manikin-based and virtual simulation training modalities.

RESULTS: Respondents were mostly 31-40 years (79, 49%) and female (127, 79%). Consultant physicians (26, 16%) and nurses (56, 35%) were in both general (98, 61%) and subspecialty (56, 35%) practice. Most had 5-10 years of experience (62, 37%) in a tertiary care setting (72, 43%). Exposure to SBT varied by profession with physicians more likely to be exposed to manikin-based (29, 30% physicians vs. 12, 19% nurses, $p<0.001$) or online training (7, 7% physician vs. 3, 5% nurses, $p<0.05$). Despite perceived barriers to SBT, respondents thought that SBT should be expanded for continuing education (84, 88% physician vs. 39, 63% nurses, $p<0.001$), teaching (73, 76% physicians vs. 16, 26% nurses, $p<0.001$), and research (65, 68% physicians vs. 14, 23% nurses, $p<0.001$). If facilities were available, nearly all respondents (92, 98% physicians; 52, 96% nurses) would recommend the use of online simulation for their center.

CONCLUSIONS: There is both need and opportunity to expand SBT beyond the current scope. Simulation Centres of Excellence should equipped for both manikin-based and virtual simulation to increase access to simulation-based education.

ARTICLE SUMMARY

Strengths and limitations of this study

- The study was a national survey of Nigerian Paediatric healthcare providers.
- A strength of the study is the inclusion of physicians and nurses practicing in both public and private secondary and tertiary healthcare facilities.
- The greater number of female respondents may underestimate exposure to computer-based and virtual reality training.
- As with limitations seen in other cross-sectional surveys, there is potential for selection and recall bias.

For peer review only - http://bmjopen.bmj.com/site/about/guidelines.xhtml

INTRODUCTION

Simulation is an approach to training that provides learners with an opportunity to practice their skills in a safe manner on a manikin or in a virtual space before a clinical encounter or procedure on a patient[1,2]. Simulation-based education is supported by adult learning theories such as the Kolb's experiential learning theory[3,4] and the Ericsson's deliberate practice theory[5] and is near the top of the Kirkpatrick triangle for supporting increased retention of knowledge and skills[6]. For this reason, elements of simulation-based education have been integrated into many global maternal and newborn health programs such as Neonatal Resuscitation Program and Helping Babies Survive[7,8].

The majority of Paediatric simulation-based training in high income countries is associated with standardized resuscitation training programs such as neonatal resuscitation program (NRP) and Paediatric advanced life support (PALS)[7]. This training is conducted in two parts using online simulation (NRP eSIM and HeartCode) and manikin-based simulation in clinical simulation facilities that are set up to mimic actual clinical settings with fixtures such as suction and gas outlets and equipment including cardiac monitors, infant warmers and hospital beds[9]. In situ simulations occur in healthcare facilities and are designed to provide convenient opportunities for practice in the healthcare setting and to identify patient safety risks[10,11].

In low-income settings, Paediatric simulation-based training in newborn resuscitation and care using the Helping Babies Survive program is conducted in non-clinical settings such as classrooms and hotel conference rooms with a low-cost manikin such as the Neonatalie manikin [Laerdal Medical] which can be filled with air or water and is resistant to adverse environmental conditions[12-15]. Refresher training is encouraged following initial training using manikins and resuscitation equipment at designated practice locations in healthcare facilities such as the Helping Babies Breathe Corner[14,15]

However, there are logistical challenges to training using simulation which involve a higher teacher to student ratio, and the need for simulation equipment and space in the clinical or educational setting for learners to be taught[16-18]. For these reasons, virtual simulations are increasingly considered as a complement to manikin-based training[19,20]. However, little is known of the access of healthcare providers in a low resource setting towards simulation-based education and, in particular, virtual reality (VR) simulation. The objective of this study was to explore the access to and utilization of various simulation modalities by in-service healthcare workers in Nigeria.

METHODS

A 35-item cross-sectional survey with questions on access to simulation-based training facilities and perceptions on simulation-based education in Paediatric settings was developed by experienced simulation educators and Paediatricians with input from Paediatric healthcare providers practicing in the U.S. and in Nigeria. The study was approved as exempt by the Seattle Children's Hospital Institutional Review Board.

For peer review only - http://bmjopen.bmj.com/site/about/guidelines.xhtml

Participants
The anonymous survey was administered on paper to a convenience sample of 200 healthcare workers who attended conference workshops conducted in January 2018 at the Paediatric Association of Nigeria Conference in Abuja (North Central), Nigeria.

Eligibility
All workshop attendees were eligible to participate in the study and were provided with a copy of the paper-based survey which included introductory study information for informed consent.

Patient and public involvement
As this was a study of healthcare providers, patients were not involved.

Measures
Access to simulation-based training facilities
Respondents were asked two questions on their access to simulation-based training facilities: “Does your institution/health facility have facilities for simulation-based training” and “Does your center have a skills-based simulation lab?”. Response options were yes or no. Respondents were asked “In what capacity does your institution use simulation-based training?” Respondents could select from three options which were not mutually exclusive: teaching, research or examination.

Exposure to simulation-based training
Respondents were asked about their awareness of and exposure to simulation-based training modalities including manikin-based, online, and virtual reality simulation. Response options were manikin-based or online training with Helping Babies Breathe(HBB), Paediatric Advanced Life Support (PALS), Essential Newborn Care (ENCC), Basic Life Support (BLS), Neonatal Resuscitation Program eSIM, HeartCode (PALS Online course), Online BLS, and Online ACLS course.

Challenges to simulation-based training
Respondents were asked questions on the challenges to having a skills-based simulation lab at their center and the challenges to online (computer-based or virtual reality) simulation. Response options on the challenges to having a skills-based simulation lab were lack of funding, lack of access to equipment, lack of curriculum, lack of space, lack of instructors trained in simulation education, and lack of awareness of an option for simulation-based training. Response options to challenges to online simulation were lack of awareness about VR based simulation, lack of internet access, lack of standardized VR training, inconsistent power supply, and lack of access to VR equipment and computers.

Perceptions of simulation-based training
Respondents were asked to identify the advantages of simulation-based training that they were aware of with response options: skills acquisition, provides feedback, step down training, monitoring and evaluation, debriefing/reflection, hands-on skills practice, teamwork/communication training, skills maintenance/retention, and examination purposes

For peer review only - http://bmjopen.bmj.com/site/about/guidelines.xhtml
For peer review only

when patients are unavailable. Respondents were asked whether simulation-based training could be expanded beyond the current scope and in what way simulation-based training should be expanded. Response options were for continued practice after initial training, teaching and research. Finally, respondents were asked whether if all facilities were available, they would recommend online simulation for their center with response options: yes or no.

Data analysis

Data were analysed using descriptive statistics and the Fisher's Exact test to examine the relationship between demographic characteristics (age, gender, profession and years in practice, type and location of practice) and respondents' access and exposure to simulation-based training facilities in their institution or healthcare facility as well as their perceptions of the benefits and challenges in using simulation-based training in their facility.

RESULTS

A total of 161 surveys were completed (RR 81%). Table 1 provides the demographic characteristics of respondents. The majority of respondents were under 40 years of age (105, 65%). Approximately one-third of respondents were nurses or nurse/midwives. There was a higher percentage of women represented (127, 79%) which is expected given the known predominance of women in the Paediatric and Nursing professions[21,22].

Table 1. Demographics of respondents

Demographic characteristics, n=161		N (%)
Age range	21 - 30 years	26 (16)
	31 - 40 years	79 (48)
	41 - 50 years	44 (27)
	> 50 years	17 (10)
Gender	Male	34 (21)
	Female	127 (79)
Profession	Consultant physician	26 (15)
	Registrar/House Officer	45 (28)
	Nurse/Nurse-midwife	62 (39)
	Medical Officer	26 (16)
	Community Health Extension Worker/Officer	9 (6)
Years of practice	< 5 years	28 (17)

For peer review only - http://bmjopen.bmj.com/site/about/guidelines.xhtml

	5 - 10 years	62 (37)
	11 - 15 years	35 (21)
	16 - 20 years	20 (12)
	> 20 years	21 (13)
Location of practice	North East	2 (1)
	North West	7(4)
	North Central (including FCT)	100 (60)
	South East	12 (7)
	South West	32(19)
	South South	14 (8)
Type of healthcare facility	Government - Tertiary care	72 (43)
	Government - Secondary care	34 (20)
	Government - Primary care	20 (12)
	Private	41 (25)
Specialty	General Paediatrics	98 (64)
	Subspecialty Paediatrics	22 (14)
	Other specialties	34 (22)

Type and location of practice
Respondents were mostly in general practice (98, 64%) with fewer in subspecialty Paediatrics (22, 14%). Most respondents had 5-10 years of experience (62, 37%) and practice a tertiary care setting (72, 43%). The majority of respondents practice in the North Central (100, 60%) or South West parts of Nigeria (32, 19%).

Access to simulation-based training facilities
Table 2 shows the distribution of respondents with simulation-based training facilities at their facility by profession, years in practice, type and location of practice. There were no differences in access to simulation-based training. Comparatively fewer respondents reported having a skills-based simulation lab at their center (22, 23% physicians vs. 21, 34% nurses, p=0.120).

For peer review only - http://bmjopen.bmj.com/site/about/guidelines.xhtml

Table 2. Access to simulation-based training in health facilities

Respondent characteristics N=155	Facilities available for simulation-based training n (%)	P-value
Profession		NS
Physician (Consultant or Registrar)	62 (66)	
Nurse	37 (61)	
Years in practice		NS
> 10 years	44 (59)	
≤ 10 years	54 (61)	
Type of facility		NS
Government	70 (61)	
Private	28 (70)	
Geographic location of practice		NS
North (North-East, North-Central, North-West Nigeria)	59 (61)	
South (South-West, South-East, South-South Nigeria)	39 (68)	

For peer review only

Exposure to simulation-based training

Where facilities were available for simulation-based training, most physicians and nurses reported the use of simulation facilities for teaching (physicians 62, 65%; nurses 34, 55%). There was low reported use for research (physicians 6, 6%; nurses 10, 16%) and examination purposes (physicians 21, 22%; nurses 6, 10%). Manikin-based training was more frequently reported than online simulation. The most reported type of training was Basic Life Support (physicians 36, 38%; nurses 18, 29%). Exposure to manikin-based training varied by profession, years in practice and level of facility. See Table 3.

Physicians were the group most likely to have been exposed to manikin-based Paediatric training programs such as Helping Babies Breathe (29, 30% physicians vs. 12, 19% nurses vs.

For peer review only - http://bmjopen.bmj.com/site/about/guidelines.xhtml

1, 11% community health workers, p<0.001) or online training in neonatal resuscitation using the NRP eSIM (7, 7% physician vs. 3, 5% nurses, p<0.05). Although the majority of physicians (91, 96%), nurses (41, 72%) owned smartphones, and many were aware that VR simulations could be run on their personal phone (43, 47% physician vs. 28, 51% nurses), only 3% (n=5) of all respondents had experienced a VR simulation.

Table 3. Exposure to manikin-based training in Basic Life Support varies by Type and Location of Facility

Basic Life Support Manikin-based training N=158	n (%)	P-value
Profession		NS
Physician (Consultant or registrar)	36 (38)	
Nurse/nurse-midwife	18 (29)	
Years in practice		NS
> 10 years	24 (32)	
< 10 years	30 (33)	
Type of facility		<0.001
Government	30 (36)	
Private	23 (58)	
Geographic location		<0.01
North (North-East, North-Central, North-West Nigeria)	25 (25)	
South (South-West, South-East, South-South Nigeria)	28 (48)	

Challenges to simulation-based training

For peer review only - http://bmjopen.bmj.com/site/about/guidelines.xhtml

Respondents identified challenges to having a skills-based simulation lab and to online (computer-based or virtual reality) simulation. There were significant differences in perceived challenges expressed by respondents from private and government facilities. See Figure 1.

Figure 1. Challenges to establishing skills-based simulation labs in private and government health facilities

Lack of awareness was the most reported challenge to using online simulation (82, 51%). Other perceived challenges to online simulation were lack of VR equipment (37, 23%) and lack of standardized VR training modules (35, 22%). Fewer respondents reported lack of internet access (24, 15%) or inconsistent power supply (21, 13%) as a challenge to online training.

Perceptions of simulation-based training

Respondents identified the advantages of simulation-based training to include skills acquisition, provides feedback, step down training, monitoring and evaluation, debriefing/reflection, hands-on skills practice, teamwork/communication training, skills maintenance/retention, and examination purposes when patients are unavailable.

Perceptions on the value of simulation-based training differed by experience. Healthcare workers with less experience were more likely to identify skills acquisition as an advantage of simulation-based training (45, 59%, > 10 years vs. 64, 71% ≤ 10 years, $p<0.05$). Healthcare workers with more than 10 years of experience were more likely to identify examination purposes when patients are unavailable (23, 30% > 10 years vs. 40, 44% ≤ 10 years, $p<0.05$), and debriefing/reflection 25, 33% > 10 years vs. 17, 19% ≤10 years) as advantages of simulation-based training. The perceived advantages of simulation also varied significantly by the profession of respondents. See Table 4.

Table 4. Perceived advantages of simulation-based training vary by profession

Advantages of simulation-based training	Physician n(%)	Nurse n(%)	p-value
Skills acquisition	83 (86)	27 (44)	<0.001
Provides feedback	47 (49)	11 (18)	<0.001
Step down training	48 (50)	21 (34)	NS
Monitoring and evaluation	47 (49)	16 (26)	<0.01

For peer review only - http://bmjopen.bmj.com/site/about/guidelines.xhtml

Debriefing/reflection	34 (35)	8 (13)	<0.01
Hands-on skills practice	64 (67)	18 (29)	<0.001
Teamwork/communication training	55 (57)	23 (37)	<0.05
Skills maintenance/retention	54 (56)	15 (24)	<0.001
Examination purposes when patients are unavailable	56 (58)	9 (15)	<0.001

All respondents thought that simulation-based training could be expanded beyond the current scope. Physicians were more likely to advocate for expanded use of simulation for continued practice after initial training (84, 88% physician vs. 39, 63% nurses, p<0.001), teaching (73, 76% physicians vs. 16, 26% nurses, p<0.001), and research (65, 68% physicians vs. 14, 23% nurses, p<0.001). If facilities were available, nearly all respondents (92, 98% physicians; 52, 96% nurses) would recommend the use of online simulation for their center.

DISCUSSION

Using data from a national survey of Paediatric healthcare workers, we found that many healthcare workers lack access to skills-based simulation labs for manikin-based training. The majority of healthcare workers own smartphones and while there is a lack of awareness of simulation-based training, the majority of survey respondents were open to expanding the use of simulations in general and online simulation in particular.

We found that less than a quarter of respondents work at institutions with skills-based simulation labs. This is in contrast with the abundance of dedicated simulation facilities in high income countries[23-25]. The exposure of healthcare workers to simulation-based training varies by profession, years of experience and type of facility. More private facility healthcare workers reported exposure to simulation-based training than respondents at government healthcare facilities. The perceived challenges to establishing skills-based simulation labs were comparatively greater for respondents at government healthcare facilities with the greatest barriers being the lack of funding and access to equipment such as manikins. Establishing dedicated, well-equipped simulation or clinical skills centres at government healthcare facilities will address these challenges along with the need to develop simulation training curricula, identify/construct new space and train simulation instructors[25].

While many of our respondents could identify advantages of simulation-based training, their responses varied by profession and experience. A variety of approaches have been described

For peer review only - http://bmjopen.bmj.com/site/about/guidelines.xhtml

for interprofessional education including role play, manikin-based and virtual simulations. Interprofessional curricula may have differing impacts on learners of different professions[26,27]. Interprofessional virtual simulations have been shown to lead to varying changes in attitudes in for students of different health professions[28]. It is therefore reasonable to infer that healthcare workers in different professions may benefit in different ways from simulation-based training.

Healthcare workers were open to the expansion of simulation for teaching, continuing education and research and supported the introduction of online simulation. Online simulation is made more feasible than manikin-based simulation in low and middle income settings by the widespread availability of mobile phones[29,30]. We confirmed a high percentage of smartphone use among healthcare workers in our study and low concern for potential barriers such as lack of internet access or inconsistent power supply. The greatest challenge to online simulation was lack of awareness. A broad grass-roots approach that engages stakeholders in training institutions, state and national ministries of health, ministries of education, industry and health professional organizations is needed for the integration of simulation-based training into continuing education programs that support the acquisition and retention of skills by in-service healthcare workers.

This study had some limitations. The data were obtained by self-report and could be subject to recall bias. Overall, women were more likely to respond to the survey. As a result, exposure to computer-based and virtual reality training may be underestimated. While physicians (both consultants and registrars) and nurses were represented in this study, other cadres of healthcare workers including community health extension workers and medical officers were not well-represented and the utilization of simulation in these groups could be a subject for future study.

CONCLUSION

The access of healthcare workers to simulation-based training has been underreported in low and middle income countries. There is both need and opportunity to expand simulation-based education beyond the current scope. Each health facility should have provision for just-in-time simulation skills training locations like “Helping Babies Breathe Corners” and clinical simulation skills labs for teaching students, registrars and for regular continuing education for inservice staff. Furthermore, Simulation Centres of Excellence should be established and equipped in all geopolitical zones of Nigeria for standardized simulation facilitator training to increase access to simulation-based education.

ACKNOWLEDGEMENTS

We would like to acknowledge the healthcare workers who participated in this study.

FUNDING

This work was supported by the Bill and Melinda Gates Foundation, grant number OPP1169873.

For peer review only - http://bmjopen.bmj.com/site/about/guidelines.xhtml

AUTHOR CONTRIBUTIONS

All authors have made substantial contributions to the planning, conduct, analyses and interpretation of findings and reporting of the work described in the article and have agreed to be accountable for all aspects of the work, its accuracy and integrity. RU is responsible for the overall content as guarantor. RU and CE formulated the study objectives and survey. IR, BE and PA assisted CE with data collection. EC assisted with data entry. CS performed statistical analysis. RU wrote the first draft of the manuscript and revised and amended it with input from all authors who also approved the final version to be published. RU is the corresponding author. The corresponding author attests that all listed authors meet authorship criteria and that no others meeting the criteria have been omitted.

COMPETING INTEREST: None declared

PATIENT CONSENT FOR PUBLICATION: Not required.

DATA AVAILABILITY STATEMENT: All data relevant to the study are included in the article.

REFERENCES

1. Sawyer T, Sierocka-Castaneda A, Chan D, Berg B, Lustik M, Thompson M. Deliberate practice using simulation improves neonatal resuscitation performance. *Simulation in Healthcare.* 2011;6(6):327-336.
2. Sawyer T, Gray MM. Procedural training and assessment of competency utilizing simulation. *Semin Perinatol.* 2016;40(7):438-446.
3. Kolb DA. Learning styles and disciplinary differences. *The modern American college.* 1981:232-255.
4. Kolb AY, Kolb DA. Learning styles and learning spaces: Enhancing experiential learning in higher education. *Academy of management learning & education.* 2005;4(2):193-212.
5. Ericsson KA. Deliberate practice and the acquisition and maintenance of expert performance in medicine and related domains. *Academic medicine.* 2004;79(10):S70-S81.
6. Smidt A, Balandin S, Sigafoos J, Reed VA. The Kirkpatrick model: A useful tool for evaluating training outcomes. *http://dxdoiorg/101080/13668250903093125.* 2009.
7. Sawyer T, Umoren RA, Gray MM. Neonatal resuscitation: advances in training and practice. *Adv Med Educ Pract.* 2017;8:11-19.
8. Ersdal HL, Singhal N, Msemo G, et al. Successful implementation of Helping Babies Survive and Helping Mothers Survive programs—An Utstein formula for newborn and maternal survival. *PloS one.* 2017;12(6):e0178073.
9. Sawyer T, Umoren R, Gray MM. Neonatal resuscitation: advances in training and practice. 2017.
10. Wetzel EA, Lang TR, Pendergrass TL, Taylor RG, Geis GL. Identification of latent safety threats using high-fidelity simulation-based training with multidisciplinary neonatology teams. *Joint Commission Journal on Quality and Patient Safety.* 2013;39(6):268-268.
11. Patterson MD, Geis GL, Falcone RA, LeMaster T, Wears RL. In situ simulation: detection of safety threats and teamwork training in a high risk emergency department. *BMJ quality & safety.* 2013;22(6):468-477.

For peer review only - http://bmjopen.bmj.com/site/about/guidelines.xhtml

12. Pearlman SA, Zern SC, Blackson T, Ciarlo JA, Mackley AB, Locke RG. Use of neonatal simulation models to assess competency in bag-mask ventilation. *J Perinatol.* 2016;36(3):242-246.
13. Hawkes GA, Malik Y, Livingstone V, Dempsey EM, Ryan CA. End user bag-mask ability and perceptions of two infant resuscitation mannequins. *Acta Paediatr.* 2016;105(3):281-285.
14. Bang A, Patel A, Bellad R, et al. Helping Babies Breathe (HBB) training: What happens to knowledge and skills over time? *BMC Pregnancy and Childbirth.* 2016;16(1):364.
15. Singhal N, Lockyer J, Fidler H, et al. Helping Babies Breathe: global neonatal resuscitation program development and formative educational evaluation. *Resuscitation.* 2012;83(1):90-96.
16. Aggarwal R, Mytton OT, Derbrew M, et al. Training and simulation for patient safety. *Quality and Safety in Health Care.* 2010;19(Suppl 2):i34.
17. Curtis MT, DiazGranados D, Feldman M. Judicious Use of Simulation Technology in Continuing Medical Education. *Journal of Continuing Education in the Health Professions.* 2012;32(4):255-260.
18. Spooner N, Hurst S, Khadra M. Medical simulation technology: Educational overview, industry leaders, and what's missing. *Hospital topics.* 2012;90(3):57-64.
19. Umoren RA PJ, Sweigart L, Rybas N, Gossett E, Johnson M, Allen M, Scott PJ, Truman B, Das RR. TeamSTEPPS Virtual Teams: Interactive Virtual Team Training and Practice for Health Professional Learners. *Creative Nursing.* 2017;23(3).
20. Umoren R, Stadler DJ, Gasior SL, Al-Sheikhly D, Truman B, Lowe C. Global collaboration and team-building through 3D virtual environments. *Innovations in Global Medical and Health Education.* 2014:1.
21. O'Lynn CE. Gender-based barriers for male students in Nursing education programs: Prevalence and perceived importance. *Journal of Nursing Education.* 2004;43(5):229-236.
22. Vassar L. How medical specialties vary by gender. *AMA Wire.* 2015;1.
23. Ziv A, Erez D, Munz Y, et al. The Israel Center for Medical Simulation: a paradigm for cultural change in medical education. *Acad Med.* 2006;81(12):1091-1097.
24. Akaike M, Fukutomi M, Nagamune M, et al. Simulation-based medical education in clinical skills laboratory. *J Med Invest.* 2012;59(1-2):28-35.
25. Lopreiato JO, Sawyer T. Simulation-based medical education in Paediatrics. *Acad Pediatr.* 2015;15(2):134-142.
26. Carpenter J, Dickinson H. *Interprofessional education and training.* policy press; 2016.
27. West C, Graham L, Palmer RT, et al. Implementation of interprofessional education (IPE) in 16 U.S. medical schools: Common practices, barriers and facilitators. *J Interprof Educ Pract.* 2016;4:41-49.
28. Umoren RA, Scott PJ, Sweigart L, et al. A comparison of teamwork attitude changes with virtual TeamSTEPPS® simulations in health professional students. *Journal of Interprofessional Education & Practice.* 2018;10:51-55.
29. Asangansi I, Braa K. The emergence of mobile-supported national health information systems in developing countries. *Stud Health Technol Inform.* 2010;160(Pt 1):540-544.
30. Bucher SL. mHBB: Using mobile phones to support Helping Babies Breathe in Kenya. In: Levine R CAKSSUGCPJHSe, ed. *mHealth Compendium.* Vol Volume 5. Arlington, VA: Management Sciences for Health; 2015: http://www.africanstrategies4health.org/uploads/1/3/5/3/13538666/mhealthvol5_final_15jun15_webv.pdf#page=56.

For peer review only - http://bmjopen.bmj.com/site/about/guidelines.xhtml

Figure Legend

Figure 1. Challenges to establishing skills-based simulation labs in private and government health facilities

Legend: *$p<0.05$

For peer review only

For peer review only - http://bmjopen.bmj.com/site/about/guidelines.xhtml

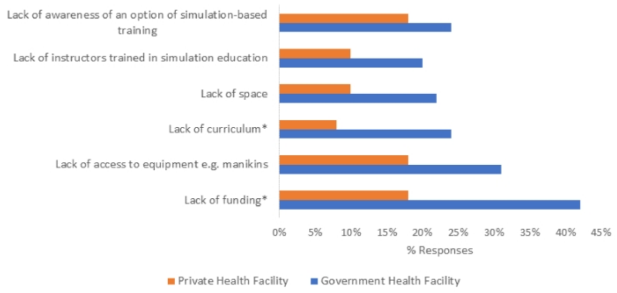

Figure 1. Challenges to establishing skills-based simulation labs in private and government health facilities
Legend: *$p<0.05$

338x190mm (300 x 300 DPI)

Reporting checklist for cross sectional study.

Based on the STROBE cross sectional guidelines.

Instructions to authors

Complete this checklist by entering the page numbers from your manuscript where readers will find each of the items listed below.

Your article may not currently address all the items on the checklist. Please modify your text to include the missing information. If you are certain that an item does not apply, please write "n/a" and provide a short explanation.

Upload your completed checklist as an extra file when you submit to a journal.

In your methods section, say that you used the STROBE cross sectionalreporting guidelines, and cite them as:

von Elm E, Altman DG, Egger M, Pocock SJ, Gotzsche PC, Vandenbroucke JP. The Strengthening the Reporting of Observational Studies in Epidemiology (STROBE) Statement: guidelines for reporting observational studies.

		Reporting Item	Page Number
**Title and abstract**			
Title	#1a	Indicate the study's design with a commonly used term in the title or the abstract	1

For peer review only

Abstract	#1b	Provide in the abstract an informative and balanced summary of what was done and what was found	1
**Introduction**			
Background / rationale	#2	Explain the scientific background and rationale for the investigation being reported	2
Objectives	#3	State specific objectives, including any prespecified hypotheses	2
**Methods**			
Study design	#4	Present key elements of study design early in the paper	2
Setting	#5	Describe the setting, locations, and relevant dates, including periods of recruitment, exposure, follow-up, and data collection	2
Eligibility criteria	#6a	Give the eligibility criteria, and the sources and methods of selection of participants.	3
	#7	Clearly define all outcomes, exposures, predictors, potential confounders, and effect modifiers. Give diagnostic criteria, if applicable	n/a
Data sources / measurement	#8	For each variable of interest give sources of data and details of methods of assessment (measurement). Describe comparability of assessment methods if there is more than one group. Give information separately for for exposed and unexposed groups if applicable.	3

For peer review only

Bias	#9	Describe any efforts to address potential sources of bias	3
Study size	#10	Explain how the study size was arrived at	n/a
Quantitative variables	#11	Explain how quantitative variables were handled in the analyses. If applicable, describe which groupings were chosen, and why	4
Statistical methods	#12a	Describe all statistical methods, including those used to control for confounding	4
Statistical methods	#12b	Describe any methods used to examine subgroups and interactions	4
Statistical methods	#12c	Explain how missing data were addressed	4
Statistical methods	#12d	If applicable, describe analytical methods taking account of sampling strategy	4
Statistical methods	#12e	Describe any sensitivity analyses	4
**Results**			
Participants	#13a	Report numbers of individuals at each stage of study—eg numbers potentially eligible, examined for eligibility, confirmed eligible, included in the study, completing follow-up, and analysed. Give information separately for for exposed and unexposed groups if applicable.	4
Participants	#13b	Give reasons for non-participation at each stage	n/a

For peer review only

Participants	#13c	Consider use of a flow diagram	n/a
Descriptive data	#14a	Give characteristics of study participants (eg demographic, clinical, social) and information on exposures and potential confounders. Give information separately for exposed and unexposed groups if applicable.	4
Descriptive data	#14b	Indicate number of participants with missing data for each variable of interest	Tables 1-4
Outcome data	#15	Report numbers of outcome events or summary measures. Give information separately for exposed and unexposed groups if applicable.	n/a
Main results	#16a	Give unadjusted estimates and, if applicable, confounder-adjusted estimates and their precision (eg, 95% confidence interval). Make clear which confounders were adjusted for and why they were included	4
Main results	#16b	Report category boundaries when continuous variables were categorized	4
Main results	#16c	If relevant, consider translating estimates of relative risk into absolute risk for a meaningful time period	4
Other analyses	#17	Report other analyses done—e.g., analyses of subgroups and interactions, and sensitivity analyses	n/a
**Discussion**			
Key results	#18	Summarise key results with reference to study objectives	6

For peer review only

Limitations	#19	Discuss limitations of the study, taking into account sources of potential bias or imprecision. Discuss both direction and magnitude of any potential bias.	7
Interpretation	#20	Give a cautious overall interpretation considering objectives, limitations, multiplicity of analyses, results from similar studies, and other relevant evidence.	7
Generalisability	#21	Discuss the generalisability (external validity) of the study results	7
**Other Information**			
Funding	#22	Give the source of funding and the role of the funders for the present study and, if applicable, for the original study on which the present article is based	1

The STROBE checklist is distributed under the terms of the Creative Commons Attribution License CC-BY. This checklist was completed on 31. August 2019 using https://www.goodreports.org/, a tool made by the EQUATOR Network in collaboration with Penelope.ai

For peer review only

BMJ Open

Perspectives on Simulation-based Training from Paediatric Healthcare Providers in Nigeria: A National Survey

Journal:	*BMJ Open*
Manuscript ID	bmjopen-2019-034029.R1
Article Type:	Original research
Date Submitted by the Author:	05-Dec-2019
Complete List of Authors:	Umoren, Rachel; University of Washington School of Medicine; Seattle Children's Hospital<br>Ezeaka, V; University of Lagos College of Medicine, Paediatrics<br>Fajolu, Ireti; University of Lagos College of Medicine, Paediatrics<br>Ezenwa, Beatrice; University of Lagos College of Medicine, Paediatrics<br>Akintan, Patricia; University of Lagos College of Medicine, Paediatrics<br>Chukwu, Emeka; University of Washington School of Medicine<br>Spiekerman, Chuck; University of Washington School of Medicine
**Primary Subject Heading**:	Paediatrics
Secondary Subject Heading:	Medical education and training
Keywords:	World Wide Web technology < BIOTECHNOLOGY & BIOINFORMATICS, EDUCATION & TRAINING (see Medical Education & Training), MEDICAL EDUCATION & TRAINING, PAEDIATRICS

SCHOLARONE™ Manuscripts

I, the Submitting Author has the right to grant and does grant on behalf of all authors of the Work (as defined in the below author licence), an exclusive licence and/or a non-exclusive licence for contributions from authors who are: i) UK Crown employees; ii) where BMJ has agreed a CC-BY licence shall apply, and/or iii) in accordance with the terms applicable for US Federal Government officers or employees acting as part of their official duties; on a worldwide, perpetual, irrevocable, royalty-free basis to BMJ Publishing Group Ltd ("BMJ") its licensees and where the relevant Journal is co-owned by BMJ to the co-owners of the Journal, to publish the Work in this journal and any other BMJ products and to exploit all rights, as set out in our licence.

The Submitting Author accepts and understands that any supply made under these terms is made by BMJ to the Submitting Author unless you are acting as an employee on behalf of your employer or a postgraduate student of an affiliated institution which is paying any applicable article publishing charge ("APC") for Open Access articles. Where the Submitting Author wishes to make the Work available on an Open Access basis (and intends to pay the relevant APC), the terms of reuse of such Open Access shall be governed by a Creative Commons licence – details of these licences and which Creative Commons licence will apply to this Work are set out in our licence referred to above.

Other than as permitted in any relevant BMJ Author's Self Archiving Policies, I confirm this Work has not been accepted for publication elsewhere, is not being considered for publication elsewhere and does not duplicate material already published. I confirm all authors consent to publication of this Work and authorise the granting of this licence.

For peer review only

Perspectives on Simulation-based Training from Paediatric Healthcare Providers in Nigeria: A National Survey

[1,2]Umoren RA, [3]Ezeaka VC, [3]Fajolu IB, [3]Ezenwa BN, [3]Akintan P, [1]Chukwu E, [1]Spiekerman, C.
[1]University of Washington, Seattle, Washington, U.S.A.; [2]Seattle Children's Hospital, Seattle, Washington, U.S.A.; [3]College of Medicine, University of Lagos, Lagos, Nigeria

Corresponding Author: Rachel A. Umoren MB.BCh, MS, FAAP. Associate Professor of Pediatrics, Department of Pediatrics, Seattle Children's Hospital & University of Washington, 1959 NE Pacific Street, Box 356320, Seattle, WA 98195. Email: rumoren@uw.edu. Phone: +1-206-543-3200.

Keywords: Education and training, Paediatrics, Medical education and training, World Wide Web technology.

Word count: 2901

For peer review only

ABSTRACT

OBJECTIVES: The objective of this study was to explore the access to and perceived utility of various simulation modalities by in-service healthcare providers in a resource-scarce setting.

SETTING: Paediatric training workshops at a national paediatric conference in Nigeria.

PARTICIPANTS: All 200 healthcare workers who attended the workshop sessions were eligible to participate. A total of 161 surveys were completed (response rate 81%).

PRIMARY AND SECONDARY OUTCOME MEASURES: A paper-based 25-item cross-sectional survey on simulation-based training (SBT) was administered to a convenience sample of healthcare workers from secondary and tertiary healthcare facilities.

RESULTS: Respondents were mostly 31-40 years (79, 49%) and female (127, 79%). Consultant physicians (26, 16%) and nurses (56, 35%) were in both general (98, 61%) and subspecialty (56, 35%) practice. Most had 5-10 years of experience (62, 37%) in a tertiary care setting (72, 43%). Exposure to SBT varied by profession with physicians more likely to be exposed to manikin-based (29, 30% physicians vs. 12, 19% nurses, $p<0.001$) or online training (7, 7% physician vs. 3, 5% nurses, $p<0.05$). Despite perceived barriers to SBT, respondents thought that SBT should be expanded for continuing education (84, 88% physician vs. 39, 63% nurses, $p<0.001$), teaching (73, 76% physicians vs. 16, 26% nurses, $p<0.001$), and research (65, 68% physicians vs. 14, 23% nurses, $p<0.001$). If facilities were available, nearly all respondents (92, 98% physicians; 52, 96% nurses) would recommend the use of online simulation for their centre.

CONCLUSIONS: The access of healthcare workers to SBT is limited in resource-scarce settings. While acknowledging the challenges, respondents identified many areas in which SBT may be useful, including skills acquisition, skills practice and communication training. Healthcare workers were open to the use of online SBT and expressed the need to expand SBT beyond the current scope for health professional training in Nigeria.

ARTICLE SUMMARY

Strengths and limitations of this study

- The study was a national survey of Nigerian paediatric healthcare professionals.
- The response rate to the survey was high.
- Physicians and nurses practicing in both public and private healthcare facilities were included in the study.
- The study compared responses from health professionals working secondary and tertiary in different parts of the country.
- As with limitations seen in other cross-sectional surveys, there is potential for selection and recall bias.

For peer review only - http://bmjopen.bmj.com/site/about/guidelines.xhtml

INTRODUCTION

Simulation is an approach to training that provides learners with an opportunity to practice their skills in a safe manner on a manikin or in a virtual space before a clinical encounter or procedure on a patient[1,2]. Simulation-based training (SBT) is supported by adult learning theories such as the Kolb's experiential learning theory[3,4] and the Ericsson's deliberate practice theory[5] and is near the top of the Kirkpatrick triangle for supporting increased retention of knowledge and skills[6]. For this reason, elements of SBT have been integrated into many global maternal and newborn health programs such as Neonatal Resuscitation Program and Helping Babies Survive[7,8].

The majority of paediatric SBT in high income countries is associated with standardized resuscitation training programs such as neonatal resuscitation program (NRP) and paediatric advanced life support (PALS)[7]. This training is conducted in two parts using online simulation (NRP eSIM and HeartCode) and manikin-based simulation in clinical simulation facilities that are set up to mimic actual clinical settings with fixtures such as suction and gas outlets and equipment including cardiac monitors, infant warmers and hospital beds[7]. In situ simulations occur in healthcare facilities and are designed to provide convenient opportunities for practice in the healthcare setting and to identify patient safety risks[9,10].

In low-income settings, paediatric SBT in newborn resuscitation and care using the Helping Babies Survive program is conducted in non-clinical settings such as classrooms and hotel conference rooms with a low-cost manikin such as the Neonatalie manikin [Laerdal Medical] which can be filled with air or water and is resistant to adverse environmental conditions[11-14]. Refresher training is encouraged following initial training using manikins and resuscitation equipment at designated practice locations in healthcare facilities such as the Helping Babies Breathe Corner[13,14]

However, there are logistical challenges to training using simulation which involve a higher teacher to student ratio, and the need for simulation equipment and space in the clinical or educational setting for learners to be taught[15-17]. For these reasons, virtual simulations are increasingly considered as a complement to manikin-based training[18,19]. However, little is known of the access of healthcare providers in a resource-scarce setting towards SBT and, in particular, virtual reality (VR) simulation. The objective of this study was to explore the access to and perceived utility of various simulation modalities by in-service healthcare providers in a resource-scarce setting.

METHODS

A 25-item cross-sectional survey was created by the investigators (RU, CE) who are simulation research collaborators from the University of Washington/Seattle Children's Hospital and the University of Lagos with questions on access to SBT facilities and perceptions on SBT in paediatric settings. Input was obtained from experienced simulation educators and healthcare professionals practicing in the U.S. and in Nigeria. The survey was piloted for clarity and ease of use among Nigerian paediatric healthcare professionals and revised based on feedback. The survey was designed to be delivered in English and intended for administration to paediatric

For peer review only - http://bmjopen.bmj.com/site/about/guidelines.xhtml

healthcare workers. See supplemental file. The study was approved as exempt by the Seattle Children's Hospital Institutional Review Board and ethics approval in Nigeria was obtained from the University of Lagos Health Research Ethics Committee

Participants

The anonymous survey was administered on paper to a convenience sample of 200 healthcare workers who attended conference workshops conducted in January 2018 at the Paediatric Association of Nigeria Conference in Abuja (North Central), Nigeria. All participants were English-speaking.

Eligibility

All workshop attendees were eligible to participate in the study and were provided with a copy of the paper-based survey which included information about study..

Patient and public involvement

As this was a study of healthcare providers, patients were not involved.

Measures

Access to SBT facilities

Respondents were asked two questions on their access to SBT facilities: "Does your institution/health facility have facilities for SBT" and "Does your centre have a skills-based simulation lab?". Response options were yes or no. Respondents were asked "In what capacity does your institution use SBT?" Respondents could select from three options which were not mutually exclusive: teaching, research or examination.

Exposure to SBT

Respondents were asked about their awareness of and exposure to SBT modalities including manikin-based, online, and virtual reality simulation. Response options were manikin-based or online training with Helping Babies Breathe(HBB), Paediatric Advanced Life Support (PALS), Essential Newborn Care (ENCC), Basic Life Support (BLS), Neonatal Resuscitation Program eSIM, HeartCode (PALS Online course), Online BLS, and Online ACLS course. No examples of virtual reality simulations specific for paediatric training were available at the time of the survey, but respondents were asked if they had ever used virtual reality simulations.

Challenges to SBT

Respondents were asked questions on the challenges to having a skills-based simulation lab at their centre and the challenges to online (computer-based or virtual reality) simulation. Response options on the challenges to having a skills-based simulation lab were lack of funding, lack of access to equipment, lack of curriculum, lack of space, lack of instructors trained in simulation education, and lack of awareness of an option for SBT. Response options to challenges to online simulation were lack of awareness about VR based simulation, lack of internet access, lack of standardized VR training, inconsistent power supply, and lack of access to VR equipment and computers.

For peer review only - http://bmjopen.bmj.com/site/about/guidelines.xhtml

Perceptions of SBT

Respondents were asked to identify the advantages of SBT that they were aware of with response options: skills acquisition, provides feedback, step down training, monitoring and evaluation, debriefing/reflection, hands-on skills practice, teamwork/communication training, skills maintenance/retention, and examination purposes when patients are unavailable. Respondents were asked whether SBT could be expanded beyond the current scope and in what way SBT should be expanded. Response options were for continued practice after initial training, teaching and research. Finally, respondents were asked whether if all facilities were available, they would recommend online simulation for their centre with response options: yes or no.

Data analysis

Data were analysed using descriptive statistics, Pearson's Chi-square test and the Fisher's Exact test to examine the relationship between demographic characteristics and respondents' access and exposure to SBT facilities in their institution or healthcare facility as well as their perceptions of the benefits and challenges in using SBT in their facility. We specifically compared the impact of demographic characteristics such as profession (physician or nurse), years in practice and type and location of practice; on access to SBT, perceived challenges of SBT and perceived utility of SBT. In some cases, subcategories of profession (e.g. Consultant physician, registrar, house officer, medical officer), years in practice and geographic location (North vs. South geopolitical zones) were collapsed for comparison due to small numbers of respondents in individual categories. No power calculation or sample size calculation was performed as the sample size was fixed, i.e. healthcare workers attending the conference. SAS 9.4 software [SAS Institute, Cary NC] was used for the analysis.

RESULTS

A total of 161 surveys were completed (response rate 81%). Table 1 provides the demographic characteristics of respondents. The majority of respondents were under 40 years of age (105, 65%). Approximately one-third of respondents were nurses or nurse/midwives. There was a higher percentage of women represented (127, 79%) which is expected given the known predominance of women in the paediatrics and nursing professions[20,21].

Table 1. Demographics of respondents

Demographic characteristics, n=161		N (%)
Age range	21 - 30 years	26 (16)
	31 - 40 years	79 (48)
	41 - 50 years	44 (27)
	> 50 years	17 (10)
Gender	Male	34 (21)

For peer review only - http://bmjopen.bmj.com/site/about/guidelines.xhtml

	Female	127 (79)
Profession	Physician	
	Consultant	26 (15)
	Registrar/House Officer	45 (28)
	Medical Officer	26 (16)
	Non-physician	
	Nurse/Nurse-midwife	62 (39)
	Community Health Extension Worker/Officer	9 (6)
Years of practice	< 5 years	28 (17)
	5 - 10 years	62 (37)
	11 - 15 years	35 (21)
	16 - 20 years	20 (12)
	> 20 years	21 (13)
Location of practice	North East	2 (1)
	North West	7(4)
	North Central*	100 (60)
	South East	12 (7)
	South West	32(19)
	South South	14 (8)
Type of healthcare facility	Government - Tertiary care	72 (43)
	Government - Secondary care	34 (20)
	Government - Primary care	20 (12)
	Private	41 (25)
Specialty	General Paediatrics	98 (64)
	Subspecialty Paediatrics	22 (14)
	Other specialties	34 (22)

*North Central: Abuja Federal Capital Territory (FCT), the capital city of Nigeria, is located in North Central Nigeria and was the location of the conference.

Type and location of practice

Respondents were mostly in general practice (98, 64%) with fewer in subspecialty paediatrics (22, 14%). Most respondents had practiced for 10 years or less (90, 54%) and many practiced

For peer review only - http://bmjopen.bmj.com/site/about/guidelines.xhtml

in a tertiary care setting (72, 43%). The majority of respondents practice in the North Central (100, 60%) or South West parts of Nigeria (32, 19%).

Access to simulation-based training facilities

Table 2 shows the distribution of respondents with SBT facilities at their facility by profession, years in practice, type and location of practice. There were no differences in access to SBT. Comparatively fewer respondents reported having a skills-based simulation lab at their centre (22, 23% physicians vs. 21, 34% nurses, p=0.120).

Table 2. Access to simulation-based training in health facilities

Respondent characteristics N=155	Facilities available for simulation-based training n (%)	P-value
**Profession**		NS
Physician	62 (66)	
Nurse	37 (61)	
**Years in practice**		NS
> 10 years	44 (62)	
≤ 10 years	54 (66)	
**Type of facility**		NS
Government	70 (61)	
Private	28 (70)	
**Geographic location of practice**		NS
North	59 (61)	
South	39 (68)	

North = North-East, North-Central, North-West Nigeria geopolitical zones
South = South-West, South-East, South-South Nigeria geopolitical zones

For peer review only

Exposure to simulation-based training

Where facilities were available for SBT, most physicians and nurses reported the use of simulation facilities for teaching (physicians 62, 65%; nurses 34, 55%). There was low reported use for research (physicians 6, 6%; nurses 10, 16%) and examination purposes (physicians 21, 22%; nurses 6, 10%). Manikin-based training was more frequently reported than online simulation. The most reported type of training was Basic Life Support (physicians 36, 38%; nurses 18, 29%). Exposure to manikin-based training varied by type of facility and geographic location (Table 3).

Physicians were the group most likely to have been exposed to manikin-based paediatric training programs such as Helping Babies Breathe (29, 30% physicians vs. 12, 19% nurses vs. 1, 11% community health workers, p<0.001) or online training in neonatal resuscitation using the NRP eSIM (7, 7% physician vs. 3, 5% nurses vs. 0, 0% community healthcare workers p<0.05). Although the majority of physicians (91, 96%) and nurses (41, 72%) owned

For peer review only - http://bmjopen.bmj.com/site/about/guidelines.xhtml

smartphones, and many were aware that VR simulations could be run on their personal phone (43, 47% physician vs. 28, 51% nurses), only 3% (n=5) of all respondents had experienced a VR simulation.

Table 3. Exposure to manikin-based training in Basic Life Support varies by Type and Location of Facility

Basic Life Support Manikin-based training N=158	n (%)	P-value
**Profession**		NS
Physician (Consultant or registrar)	36 (38)	
Nurse/nurse-midwife	18 (29)	
**Years in practice**		NS
> 10 years	24 (32)	
< 10 years	30 (33)	
**Type of facility**		<0.001
Government	30 (36)	
Private	23 (58)	
**Geographic location**		<0.01
North	25 (25)	
South	28 (48)	

North = North-East, North-Central, North-West Nigeria geopolitical zones
South = South-West, South-East, South-South Nigeria geopolitical zones

Challenges to simulation-based training
Respondents identified challenges to having a skills-based simulation lab and to online (computer-based or virtual reality) simulation. Lack of curriculum and lack of funding were perceived as less of a barrier to establishing a skills-based simulation lab by respondents from private healthcare facilities compared with respondents from government facilities (p<0.05) (Figure 1).

Figure 1. Challenges to establishing skills-based simulation labs in private and government health facilities

Lack of awareness was the most reported challenge to using online simulation (82, 51%). Other perceived challenges to online simulation were lack of VR equipment (37, 23%) and lack of standardized VR training modules (35, 22%). Fewer respondents reported lack of internet access (24, 15%) or inconsistent power supply (21, 13%) as a challenge to online training.

For peer review only - http://bmjopen.bmj.com/site/about/guidelines.xhtml

Perceptions of simulation-based training

Respondents identified the advantages of SBT to include skills acquisition, provides feedback, step down training, monitoring and evaluation, debriefing/reflection, hands-on skills practice, teamwork/communication training, skills maintenance/retention, and examination purposes when patients are unavailable.

Perceptions on the value of SBT differed by experience. Healthcare workers with less experience were more likely to identify skills acquisition as an advantage of SBT (45, 59%, > 10 years vs. 64, 71% ≤ 10 years, $p<0.05$). Healthcare workers with less than or equal to 10 years of experience were more likely to identify examination purposes when patients are unavailable (23, 30% > 10 years vs. 40, 44% ≤ 10 years, $p<0.05$), while those with more than 10 years of experience identified debriefing/reflection (25, 33% > 10 years vs. 17, 19% ≤10 years) as advantages of SBT. The perceived advantages of simulation also varied significantly by the profession of respondents. See Table 4.

Table 4. Perceived advantages of simulation-based training vary by profession

Advantages of simulation-based training	Physician n (%)	Nurse n (%)	p-value
Skills acquisition	83 (86)	27 (44)	<0.001
Provides feedback	47 (49)	11 (18)	<0.001
Step down training	48 (50)	21 (34)	NS
Monitoring and evaluation	47 (49)	16 (26)	<0.01
Debriefing/reflection	34 (35)	8 (13)	<0.01
Hands-on skills practice	64 (67)	18 (29)	<0.001
Teamwork/communication training	55 (57)	23 (37)	<0.05
Skills maintenance/retention	54 (56)	15 (24)	<0.001
Examination purposes when patients are unavailable	56 (58)	9 (15)	<0.001

All respondents thought that SBT could be expanded beyond the current scope. Physicians were more likely to advocate for expanded use of simulation for continued practice after initial training (84, 88% physician vs. 39, 63% nurses, $p<0.001$). They were also more likely to advocate for simulation for teaching (73, 76% physicians vs. 16, 26% nurses, $p<0.001$), and

For peer review only - http://bmjopen.bmj.com/site/about/guidelines.xhtml

research (65, 68% physicians vs. 14, 23% nurses, p<0.001). If facilities were available, nearly all respondents (92, 98% physicians; 52, 96% nurses) would recommend the use of online simulation for their centre.

DISCUSSION

Using data from a national survey of paediatric healthcare workers, we explored the access to and perceived utility of various simulation modalities in a resource-scarce setting. Our study found that many healthcare workers lack access to skills-based simulation labs for manikin-based training. The perceived challenges to establishing skills-based simulation labs were comparatively greater for respondents at government healthcare facilities with the greatest identified barriers being the lack of funding and access to equipment such as manikins. This is in contrast with the abundance of dedicated simulation facilities in high income countries[22-24]. Dedicated spaces and equipment for SBT are only the first step, there is also a need to develop locally relevant simulation cases and to train simulation instructors in the techniques of simulation facilitation and debriefing[24,25].

The perceived utility of SBT may vary by profession and setting. While many of our respondents identified specific ways in which SBT could be used, their responses varied by profession and experience. A variety of approaches have been described for interprofessional education including role play, manikin-based and virtual simulations. Interprofessional curricula may have differing impacts on learners of different professions[26-28]. Interprofessional virtual simulations have been shown to lead to varying changes in attitudes in for students of different health professions[28]. It is therefore reasonable to infer that healthcare workers in different professions may benefit from SBT in different ways.

Healthcare workers were open to the expansion of simulation for teaching, continuing education and research and supported the introduction of online SBT. Online SBT is made more feasible than manikin-based simulation in resource-scarce settings by the widespread availability of mobile phones[29]. We confirmed a high percentage of smartphone use among healthcare workers in our study and low concern for potential barriers such as lack of internet access or inconsistent power supply. The integration of SBT into medical and nursing school curricula provides early exposure to SBT[24]. Establishing simulation programs at public and private healthcare facilities would enable the development of contextually appropriate simulation curricula and instructor courses in simulation facilitation, debriefing and research[22,23].

A broad grass-roots approach that engages stakeholders in training institutions, state and national ministries of health, ministries of education, industry and health professional organizations is needed to support the integration of SBT into pre-service training and continuing education programs for in-service healthcare workers. Continuing education programs support the acquisition and retention of skills after initial training and have been important sources of sustainable funding for SBT in high income settings[7,13-16]. These mechanisms may be leveraged to support SBT in resource-scarce settings.

For peer review only - http://bmjopen.bmj.com/site/about/guidelines.xhtml

This study had some limitations. This was a cross-sectional survey; the data were obtained by self-report and could be subject to selection and recall bias. The survey was administered to attendees at a national paediatric conference. Although respondents worked at both training and non-training institutions and in both public and private settings, their attendance at the conference may indicate that they may be more likely to be supportive of academic pursuits, including SBT. While physicians (both consultants and registrars) and nurses were represented in this study, other cadres of healthcare workers including community health extension workers and medical officers were not well-represented and the utilization of simulation in these groups could be a subject for future study.

CONCLUSION

The access of healthcare workers to SBT is limited in resource-scarce settings. However, respondents identified many areas in which SBT has utility including skills acquisition, hands-on skills practice and communication training. Lack of awareness, access to equipment and funding were identified as challenges to SBT. Healthcare workers were open to the use of online SBT and expressed the need to expand SBT beyond the current scope for pre-service and in-service health professional training in Nigeria.

ACKNOWLEDGEMENTS

We would like to acknowledge the healthcare workers who participated in this study.

FUNDING

This work was supported by the Bill and Melinda Gates Foundation, grant number OPP1169873.

AUTHOR CONTRIBUTIONS

All authors have made substantial contributions to the planning, conduct, analyses and interpretation of findings and reporting of the work described in the article and have agreed to be accountable for all aspects of the work, its accuracy and integrity. RU is responsible for the overall content as guarantor. RU and CE formulated the study objectives and survey. IF, BE and PA assisted CE with data collection. EC assisted with data entry. CS performed statistical analysis. RU wrote the first draft of the manuscript and revised and amended it with input from all authors who also approved the final version to be published. RU is the corresponding author. The corresponding author attests that all listed authors meet authorship criteria and that no others meeting the criteria have been omitted.

COMPETING INTEREST: None declared

PATIENT CONSENT FOR PUBLICATION: Not required.

DATA AVAILABILITY STATEMENT: All data relevant to the study are included in the article.

For peer review only - http://bmjopen.bmj.com/site/about/guidelines.xhtml

REFERENCES

1. Sawyer T, Sierocka-Castaneda A, Chan D, Berg B, Lustik M, Thompson M. Deliberate practice using simulation improves neonatal resuscitation performance. *Simulation in Healthcare.* 2011;6(6):327-336.
2. Sawyer T, Gray MM. Procedural training and assessment of competency utilizing simulation. *Semin Perinatol.* 2016;40(7):438-446.
3. Kolb DA. Learning styles and disciplinary differences. *The modern American college.* 1981:232-255.
4. Kolb AY, Kolb DA. Learning styles and learning spaces: Enhancing experiential learning in higher education. *Academy of management learning & education.* 2005;4(2):193-212.
5. Ericsson KA. Deliberate practice and the acquisition and maintenance of expert performance in medicine and related domains. *Academic medicine.* 2004;79(10):S70-S81.
6. Smidt A, Balandin S, Sigafoos J, Reed VA. The Kirkpatrick model: A useful tool for evaluating training outcomes. *http://dxdoiorg/101080/13668250903093125.* 2009.
7. Sawyer T, Umoren RA, Gray MM. Neonatal resuscitation: advances in training and practice. *Adv Med Educ Pract.* 2017;8:11-19.
8. Ersdal HL, Singhal N, Msemo G, et al. Successful implementation of Helping Babies Survive and Helping Mothers Survive programs—An Utstein formula for newborn and maternal survival. *PloS one.* 2017;12(6):e0178073.
9. Wetzel EA, Lang TR, Pendergrass TL, Taylor RG, Geis GL. Identification of latent safety threats using high-fidelity simulation-based training with multidisciplinary neonatology teams. *Joint Commission Journal on Quality and Patient Safety.* 2013;39(6):268-268.
10. Patterson MD, Geis GL, Falcone RA, LeMaster T, Wears RL. In situ simulation: detection of safety threats and teamwork training in a high risk emergency department. *BMJ quality & safety.* 2013;22(6):468-477.
11. Pearlman SA, Zern SC, Blackson T, Ciarlo JA, Mackley AB, Locke RG. Use of neonatal simulation models to assess competency in bag-mask ventilation. *J Perinatol.* 2016;36(3):242-246.
12. Hawkes GA, Malik Y, Livingstone V, Dempsey EM, Ryan CA. End user bag-mask ability and perceptions of two infant resuscitation mannequins. *Acta Paediatr.* 2016;105(3):281-285.
13. Bang A, Patel A, Bellad R, et al. Helping Babies Breathe (HBB) training: What happens to knowledge and skills over time? *BMC Pregnancy and Childbirth.* 2016;16(1):364.
14. Singhal N, Lockyer J, Fidler H, et al. Helping Babies Breathe: global neonatal resuscitation program development and formative educational evaluation. *Resuscitation.* 2012;83(1):90-96.
15. Aggarwal R, Mytton OT, Derbrew M, et al. Training and simulation for patient safety. *Quality and Safety in Health Care.* 2010;19(Suppl 2):i34.
16. Curtis MT, DiazGranados D, Feldman M. Judicious Use of Simulation Technology in Continuing Medical Education. *Journal of Continuing Education in the Health Professions.* 2012;32(4):255-260.
17. Spooner N, Hurst S, Khadra M. Medical simulation technology: Educational overview, industry leaders, and what's missing. *Hospital topics.* 2012;90(3):57-64.
18. Umoren RA PJ, Sweigart L, Rybas N, Gossett E, Johnson M, Allen M, Scott PJ, Truman B, Das RR. TeamSTEPPS Virtual Teams: Interactive Virtual Team Training and Practice for Health Professional Learners. *Creative Nursing.* 2017;23(3).

For peer review only - http://bmjopen.bmj.com/site/about/guidelines.xhtml

19. Umoren R, Stadler DJ, Gasior SL, Al-Sheikhly D, Truman B, Lowe C. Global collaboration and team-building through 3D virtual environments. *Innovations in Global Medical and Health Education.* 2014:1.
20. O'Lynn CE. Gender-based barriers for male students in Nursing education programs: Prevalence and perceived importance. *Journal of Nursing Education.* 2004;43(5):229-236.
21. Vassar L. How medical specialties vary by gender. *AMA Wire.* 2015;1.
22. Ziv A, Erez D, Munz Y, et al. The Israel Center for Medical Simulation: a paradigm for cultural change in medical education. *Acad Med.* 2006;81(12):1091-1097.
23. Akaike M, Fukutomi M, Nagamune M, et al. Simulation-based medical education in clinical skills laboratory. *J Med Invest.* 2012;59(1-2):28-35.
24. Lopreiato JO, Sawyer T. Simulation-based medical education in Pediatrics. *Acad Pediatr.* 2015;15(2):134-142.
25. Sawyer T, Eppich W, Brett-Fleegler M, Grant V, Cheng A. More Than One Way to Debrief. *Simul Healthc.* 2016;11(3):209-217.
26. Carpenter J, Dickinson H. *Interprofessional education and training.* policy press; 2016.
27. West C, Graham L, Palmer RT, et al. Implementation of interprofessional education (IPE) in 16 U.S. medical schools: Common practices, barriers and facilitators. *J Interprof Educ Pract.* 2016;4:41-49.
28. Umoren RA, Scott PJ, Sweigart L, et al. A comparison of teamwork attitude changes with virtual TeamSTEPPS® simulations in health professional students. *Journal of Interprofessional Education & Practice.* 2018;10:51-55.
29. Asangansi I, Braa K. The emergence of mobile-supported national health information systems in developing countries. *Stud Health Technol Inform.* 2010;160(Pt 1):540-544.

Figure Legend

Figure 1. Challenges to establishing skills-based simulation labs in private and government health facilities

Legend: *p<0.05

For peer review only

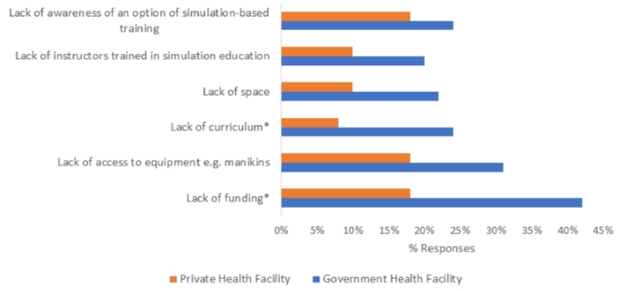

Figure 1. Challenges to establishing skills-based simulation labs in private and government health facilities
Legend: *p<0.05

338x190mm (300 x 300 DPI)

Survey on simulation based learning/practice

Please answer the following questions:

1. **What is your age range?**
   a. <21 **b.** 21-30 **c.** 31-40 **d.** 41-50 **e.** >50
2. **Sex**
   a. Male b. Female
3. **Profession**
   a. Consultant physician **b.** Senior resident/registrar **c.** Resident/registrar **d.** Nurse **e.** Other __________
4. **Years of practice**
   a. < 5 years **b.** 5-10 years **c.** 11-15 years **d.** 16-20 years **e.** >20 years
5. **Current location of practice**
   ____
6. **Type of practice**
   a. Government facility
      i. Tertiary care
      ii. Secondary care
      iii. Primary care
   b. Private practice
   c. Faith-based facility
7. **Specialty**
   a. General Pediatrics
   b. Subspecialty Pediatrics (Please specify ____________________)
   c. Other specialty (Please specify ____________________)
8. **Are you aware of the use of simulation-based training at your institution?**
   a. Yes **b.** No
9. **If yes in Q8, in what capacity does your institution use simulation-based training? (select all that apply)**
   a. Teaching **b.** Research **c.** Examination
10. **Does your center have a skills-based simulation lab?**
    a. Yes **b**. No
11. **If yes in Q10, what is the skills-based simulation lab available for? (select all that apply)**
    a. Skills practice e.g. HBB Newborn corner **b.** Teaching **c.** Research **d.** Examination
12. **If no in Q10, what are the challenges to having a skills-based simulation lab at your center?**
    a. Lack of funding

For peer review only

b. Lack of access to equipment e.g. manikins
c. Lack of curriculum
d. Lack of space
e. Lack of instructors trained in simulation education
f. Lack of awareness of an option for simulation-based training

13. Which modality of simulation based training have you been exposed to? (select all that apply)

a. Manikin-based training
   i. HBB
   ii. NRT
   iii. PALS
   iv. ENCC
   v. BLS
b. Online (computer-based) simulation
   i. NRP eSIM™
   ii. HeartCode™ (PALS online course)
   iii. Online Basic Life Support course
   iv. Online ACLS course

14. Are you aware of virtual reality simulation training?

a. Yes b. No

15. If yes in Q14, when or where were you exposed to virtual reality simulation?

---

16. If no in Q14, what are the challenges to online (computer-based or virtual reality) simulation?

a. Lack of internet access
b. Lack of standardized VR training modules
c. Inconsistent power supply
d. Lack of access to VR equipment and computers

17. Which of these advantages of simulation-based training are you aware of (select all that apply)?

a. Skills acquisition
b. Provides feedback
c. Step down training
d. Monitoring and evaluation
e. Debriefing/reflection
f. Hands-on skills practice
g. Teamwork/communication training
h. Skills maintenance/retention
i. Examination purposes when patients are unavailable

18. What type of mobile phone device do you own or use? (Choose all that apply)

a. Tablet (Eg. Ipad, tablets)

For peer review only - http://bmjopen.bmj.com/site/about/guidelines.xhtml

b. Smart Phone (eg. Iphone, Samsung, Techno, Nexus, Infinix etc.)
c. Feature phone (Eg. Does some gprs based activities)
d. Basic (Eg. Used for call and SMS only)

19. What is the manufacturer and model of your phone/mobile device?

____

20. If you are using an android enabled device, what android version does your device run (To find out, Goto |Settings->General->AboutDevice| and look for version number)
a. Gingerbread (version 2.3)
b. Ice Cream Sandwich (version 4.0)
c. Jelly Bean (version 4.1 – 4.3)
d. KitKat (Version 4.4)
e. Lollipop (Version 5.0 – 5.1)
f. Marshmallow (Version 6.0)
g. Nougat (Version 7)
h. Oreo (Version 8)

21. Do you use mobile device currently for your work?
a. Yes **b.** No

22. Do you think simulation based training could be expanded beyond the current scope?
a. Yes **b.** No

23. If yes in Q22, in what way should simulation-based training be expanded in Nigeria?
a. Continued practice after initial training
b. Teaching
c. Research

24. If all facilities were available, would you recommend online simulation for your center?
a. Yes b. No

25. If No in Q24, please state your reason(s)____

For peer review only

Reporting checklist for cross sectional study.

Based on the STROBE cross sectional guidelines.

Instructions to authors

Complete this checklist by entering the page numbers from your manuscript where readers will find each of the items listed below.

Your article may not currently address all the items on the checklist. Please modify your text to include the missing information. If you are certain that an item does not apply, please write "n/a" and provide a short explanation.

Upload your completed checklist as an extra file when you submit to a journal.

In your methods section, say that you used the STROBE cross sectionalreporting guidelines, and cite them as:

von Elm E, Altman DG, Egger M, Pocock SJ, Gotzsche PC, Vandenbroucke JP. The Strengthening the Reporting of Observational Studies in Epidemiology (STROBE) Statement: guidelines for reporting observational studies.

For peer review only

		Reporting Item	Page Number
**Title and abstract**			
Title	#1a	Indicate the study's design with a commonly used term in the title or the abstract	1

Abstract	#1b	Provide in the abstract an informative and balanced summary of what was done and what was found	1
**Introduction**			
Background / rationale	#2	Explain the scientific background and rationale for the investigation being reported	2
Objectives	#3	State specific objectives, including any prespecified hypotheses	2
**Methods**			
Study design	#4	Present key elements of study design early in the paper	2
Setting	#5	Describe the setting, locations, and relevant dates, including periods of recruitment, exposure, follow-up, and data collection	2
Eligibility criteria	#6a	Give the eligibility criteria, and the sources and methods of selection of participants.	3
	#7	Clearly define all outcomes, exposures, predictors, potential confounders, and effect modifiers. Give diagnostic criteria, if applicable	n/a
Data sources / measurement	#8	For each variable of interest give sources of data and details of methods of assessment (measurement). Describe comparability of assessment methods if there is more than one group. Give information separately for for exposed and unexposed groups if applicable.	3

For peer review only

Bias	#9	Describe any efforts to address potential sources of bias	3
Study size	#10	Explain how the study size was arrived at	n/a
Quantitative variables	#11	Explain how quantitative variables were handled in the analyses. If applicable, describe which groupings were chosen, and why	4
Statistical methods	#12a	Describe all statistical methods, including those used to control for confounding	4
Statistical methods	#12b	Describe any methods used to examine subgroups and interactions	4
Statistical methods	#12c	Explain how missing data were addressed	4
Statistical methods	#12d	If applicable, describe analytical methods taking account of sampling strategy	4
Statistical methods	#12e	Describe any sensitivity analyses	4
**Results**			
Participants	#13a	Report numbers of individuals at each stage of study—eg numbers potentially eligible, examined for eligibility, confirmed eligible, included in the study, completing follow-up, and analysed. Give information separately for for exposed and unexposed groups if applicable.	4
Participants	#13b	Give reasons for non-participation at each stage	n/a

For peer review only

Participants	#13c	Consider use of a flow diagram	n/a
Descriptive data	#14a	Give characteristics of study participants (eg demographic, clinical, social) and information on exposures and potential confounders. Give information separately for exposed and unexposed groups if applicable.	4
Descriptive data	#14b	Indicate number of participants with missing data for each variable of interest	Tables 1-4
Outcome data	#15	Report numbers of outcome events or summary measures. Give information separately for exposed and unexposed groups if applicable.	n/a
Main results	#16a	Give unadjusted estimates and, if applicable, confounder-adjusted estimates and their precision (eg, 95% confidence interval). Make clear which confounders were adjusted for and why they were included	4
Main results	#16b	Report category boundaries when continuous variables were categorized	4
Main results	#16c	If relevant, consider translating estimates of relative risk into absolute risk for a meaningful time period	4
Other analyses	#17	Report other analyses done—e.g., analyses of subgroups and interactions, and sensitivity analyses	n/a
**Discussion**			
Key results	#18	Summarise key results with reference to study objectives	6

For peer review only

Limitations	#19	Discuss limitations of the study, taking into account sources of potential bias or imprecision. Discuss both direction and magnitude of any potential bias.	7
Interpretation	#20	Give a cautious overall interpretation considering objectives, limitations, multiplicity of analyses, results from similar studies, and other relevant evidence.	7
Generalisability	#21	Discuss the generalisability (external validity) of the study results	7
**Other Information**			
Funding	#22	Give the source of funding and the role of the funders for the present study and, if applicable, for the original study on which the present article is based	1

The STROBE checklist is distributed under the terms of the Creative Commons Attribution License CC-BY. This checklist was completed on 31. August 2019 using https://www.goodreports.org/, a tool made by the EQUATOR Network in collaboration with Penelope.ai

For peer review only

BMJ Open

Perspectives on Simulation-based Training from Paediatric Healthcare Providers in Nigeria: A National Survey

Journal:	*BMJ Open*
Manuscript ID	bmjopen-2019-034029.R2
Article Type:	Original research
Date Submitted by the Author:	21-Jan-2020
Complete List of Authors:	Umoren, Rachel; University of Washington School of Medicine; Seattle Children's Hospital<br>Ezeaka, V; University of Lagos College of Medicine, Paediatrics<br>Fajolu, Ireti; University of Lagos College of Medicine, Paediatrics<br>Ezenwa, Beatrice; University of Lagos College of Medicine, Paediatrics<br>Akintan, Patricia; University of Lagos College of Medicine, Paediatrics<br>Chukwu, Emeka; University of Washington School of Medicine<br>Spiekerman, Chuck; University of Washington School of Medicine
**Primary Subject Heading**:	Paediatrics
Secondary Subject Heading:	Medical education and training
Keywords:	World Wide Web technology < BIOTECHNOLOGY & BIOINFORMATICS, EDUCATION & TRAINING (see Medical Education & Training), MEDICAL EDUCATION & TRAINING, PAEDIATRICS

SCHOLARONE™
Manuscripts

I, the Submitting Author has the right to grant and does grant on behalf of all authors of the Work (as defined in the below author licence), an exclusive licence and/or a non-exclusive licence for contributions from authors who are: i) UK Crown employees; ii) where BMJ has agreed a CC-BY licence shall apply, and/or iii) in accordance with the terms applicable for US Federal Government officers or employees acting as part of their official duties; on a worldwide, perpetual, irrevocable, royalty-free basis to BMJ Publishing Group Ltd ("BMJ") its licensees and where the relevant Journal is co-owned by BMJ to the co-owners of the Journal, to publish the Work in this journal and any other BMJ products and to exploit all rights, as set out in our licence.

The Submitting Author accepts and understands that any supply made under these terms is made by BMJ to the Submitting Author unless you are acting as an employee on behalf of your employer or a postgraduate student of an affiliated institution which is paying any applicable article publishing charge ("APC") for Open Access articles. Where the Submitting Author wishes to make the Work available on an Open Access basis (and intends to pay the relevant APC), the terms of reuse of such Open Access shall be governed by a Creative Commons licence – details of these licences and which Creative Commons licence will apply to this Work are set out in our licence referred to above.

Other than as permitted in any relevant BMJ Author's Self Archiving Policies, I confirm this Work has not been accepted for publication elsewhere, is not being considered for publication elsewhere and does not duplicate material already published. I confirm all authors consent to publication of this Work and authorise the granting of this licence.

For peer review only

Perspectives on Simulation-based Training from Paediatric Healthcare Providers in Nigeria: A National Survey

[1,2]Umoren RA, [3]Ezeaka VC, [3]Fajolu IB, [3]Ezenwa BN, [3]Akintan P, [1]Chukwu E, [1]Spiekerman, C.
[1]University of Washington, Seattle, Washington, U.S.A.; [2]Seattle Children's Hospital, Seattle, Washington, U.S.A.; [3]College of Medicine, University of Lagos, Lagos, Nigeria

Corresponding Author: Rachel A. Umoren MB.BCh, MS, FAAP. Associate Professor of Pediatrics, Department of Pediatrics, Seattle Children's Hospital & University of Washington, 1959 NE Pacific Street, Box 356320, Seattle, WA 98195. Email: rumoren@uw.edu. Phone: +1-206-543-3200.

Keywords: Education and training, Paediatrics, Medical education and training, World Wide Web technology.

Word count: 2799

For peer review only - http://bmjopen.bmj.com/site/about/guidelines.xhtml

ABSTRACT

OBJECTIVES: The objective of this study was to explore the access to, and perceived utility of, various simulation modalities by in-service healthcare providers in a resource-scarce setting.

SETTING: Paediatric training workshops at a national paediatric conference in Nigeria.

PARTICIPANTS: All 200 healthcare workers who attended the workshop sessions were eligible to participate. A total of 161 surveys were completed (response rate 81%).

PRIMARY AND SECONDARY OUTCOME MEASURES: A paper-based 25-item cross-sectional survey on simulation-based training (SBT) was administered to a convenience sample of healthcare workers from secondary and tertiary healthcare facilities.

RESULTS: Respondents were mostly 31-40 years of age (79, 49%) and female (127, 79%). Consultant physicians (26, 16%) and nurses (56, 35%) were in both general (98, 61%) and subspecialty (56, 35%) practice. Most had 5-10 years of experience (62, 37%) in a tertiary care setting (72, 43%). Exposure to SBT varied by profession with physicians more likely to be exposed to manikin-based (29, 30% physicians vs. 12, 19% nurses, $p<0.001$) or online training (7, 7% physician vs. 3, 5% nurses, $p<0.05$). Despite perceived barriers to SBT, respondents thought that SBT should be expanded for continuing education (84, 88% physician vs. 39, 63% nurses, $p<0.001$), teaching (73, 76% physicians vs. 16, 26% nurses, $p<0.001$), and research (65, 68% physicians vs. 14, 23% nurses, $p<0.001$). If facilities were available, nearly all respondents (92, 98% physicians; 52, 96% nurses) would recommend the use of online simulation for their centre.

CONCLUSIONS: The access of healthcare workers to SBT is limited in resource-scarce settings. While acknowledging the challenges, respondents identified many areas in which SBT may be useful, including skills acquisition, skills practice and communication training. Healthcare workers were open to the use of online SBT and expressed the need to expand SBT beyond the current scope for health professional training in Nigeria.

ARTICLE SUMMARY

Strengths and limitations of this study

- The study was a national survey of Nigerian paediatric healthcare professionals.
- The response rate to the survey was high.
- Physicians and nurses practicing in both public and private healthcare facilities were included in the study.
- The study compared responses from health professionals working secondary and tertiary working in different parts of the country.
- As with limitations seen in other cross-sectional surveys, there is potential for selection and recall bias.

For peer review only - http://bmjopen.bmj.com/site/about/guidelines.xhtml

INTRODUCTION

Simulation is an approach to training that provides learners with an opportunity to practice their skills in a safe manner on a manikin or in a virtual space before a clinical encounter or procedure on a patient[1,2]. Simulation-based training (SBT) is supported by adult learning theories such as the Kolb's experiential learning theory[3,4] and the Ericsson's deliberate practice theory[5] and is near the top of the Kirkpatrick triangle for supporting increased retention of knowledge and skills[6]. For this reason, elements of SBT have been integrated into many global maternal and newborn health programs such as Neonatal Resuscitation Program and Helping Babies Survive[7,8].

The majority of paediatric SBT in high income countries is associated with standardized resuscitation training programs such as neonatal resuscitation program (NRP) and paediatric advanced life support (PALS)[7]. This training is conducted in two parts using online simulation (NRP eSIM and HeartCode) and manikin-based simulation in clinical simulation facilities that are set up to mimic actual clinical settings with fixtures such as suction and gas outlets and equipment including cardiac monitors, infant warmers and hospital beds[7]. In situ simulations occur in healthcare facilities and are designed to provide convenient opportunities for practice in the healthcare setting and to identify patient safety risks[9,10].

In low-income settings, paediatric SBT in newborn resuscitation and care using the Helping Babies Survive program is conducted in non-clinical settings such as classrooms and hotel conference rooms with a low-cost manikin such as the Neonatalie manikin [Laerdal Medical] which can be filled with air or water and is resistant to adverse environmental conditions[11-14]. Refresher training is encouraged following initial training using manikins and resuscitation equipment at designated practice locations in healthcare facilities such as the Helping Babies Breathe Corner[13,14]

However, there are logistical challenges to training using simulation which involve a higher teacher to student ratio, and the need for simulation equipment and space in the clinical or educational setting for learners to be taught[15-17]. For these reasons, virtual simulations are increasingly considered as a complement to manikin-based training[18,19]. However, little is known of the access of healthcare providers in a resource-scarce setting towards SBT and, in particular, virtual reality (VR) simulation. The objective of this study was to explore the access to and perceived utility of various simulation modalities by in-service healthcare providers in a resource-scarce setting.

METHODS

A 25-item cross-sectional survey was created by the investigators (RU, CE) who are simulation research collaborators from the University of Washington/Seattle Children's Hospital and the University of Lagos with questions on access to SBT facilities and perceptions on SBT in paediatric settings. See supplemental file. Input was obtained from experienced simulation educators and healthcare professionals practicing in the U.S. and in Nigeria. The survey was piloted for clarity and ease of use among Nigerian paediatric healthcare professionals and revised based on feedback. The survey was designed to be delivered in English and intended

For peer review only - http://bmjopen.bmj.com/site/about/guidelines.xhtml

for administration to paediatric healthcare workers. The study was approved as exempt by the Seattle Children's Hospital Institutional Review Board and ethics approval in Nigeria was obtained from the University of Lagos Health Research Ethics Committee

Participants

The anonymous survey was administered on paper to a convenience sample of 200 healthcare workers who attended conference workshops conducted in January 2018 at the Paediatric Association of Nigeria Conference in Abuja (North Central), Nigeria. All participants were English-speaking.

Eligibility

All workshop attendees were eligible to participate in the study and were provided with a copy of the paper-based survey which included information about study.

Patient and public involvement

As this was a study of healthcare providers, patients were not involved.

Measures

Access to SBT facilities

Respondents were asked two questions on their access to SBT facilities: "Does your institution/health facility have facilities for SBT" and "Does your centre have a skills-based simulation lab?". Respondents were asked "In what capacity does your institution use SBT?" Respondents could select from three options which were not mutually exclusive: teaching, research or examination.

Exposure to SBT

Respondents were asked about their awareness of and exposure to SBT modalities including manikin-based, online, and virtual reality simulation. Within the exposure domain, no examples of virtual reality simulations specific to pediatric training were available at the time of the survey, but respondents were asked if they had ever used virtual reality simulation.

Challenges to SBT

Respondents were asked questions on the challenges to having a skills-based simulation lab at their centre and the challenges to online (computer-based or virtual reality) simulation. Response options on the challenges to having a skills-based simulation lab were lack of funding, lack of access to equipment, lack of curriculum, lack of space, lack of instructors trained in simulation education, and lack of awareness of an option for SBT.

Perceptions of SBT

Respondents were asked to identify the advantages of SBT that they were aware of, whether SBT could be expanded beyond the current scope and in what way SBT should be expanded. Finally, respondents were asked whether if all facilities were available, they would recommend online simulation for their centre with response options: yes or no.

For peer review only - http://bmjopen.bmj.com/site/about/guidelines.xhtml

Data analysis

Data were analysed using descriptive statistics, Pearson's Chi-square test and the Fisher's Exact test to examine the relationship between demographic characteristics and respondents' access and exposure to SBT facilities in their institution or healthcare facility as well as their perceptions of the benefits and challenges in using SBT in their facility. We specifically compared the impact of demographic characteristics such as profession (physician or nurse), years in practice and type and location of practice; on access to SBT, perceived challenges of SBT and perceived utility of SBT. In some cases, subcategories of profession (e.g. Consultant physician, registrar, house officer, medical officer), years in practice and geographic location (North vs. South geopolitical zones) were collapsed for comparison due to small numbers of respondents in individual categories. No power calculation or sample size calculation was performed as the sample size was fixed, i.e. healthcare workers attending the conference. SAS 9.4 software [SAS Institute, Cary NC] was used for the analysis.

RESULTS

A total of 161 surveys were completed (response rate 81%). Table 1 provides the demographic characteristics of respondents. The majority of respondents were under 40 years of age (105, 65%). Approximately one-third of respondents were nurses or nurse/midwives. There was a higher percentage of women represented (127, 79%) which is expected given the known predominance of women in the paediatrics and nursing professions[20,21].

Table 1. Demographics of respondents

Demographic characteristics, n=161			N (%)
Age range		21 - 30 years	26 (16)
		31 - 40 years	79 (48)
		41 - 50 years	44 (27)
		> 50 years	17 (10)
Gender		Male	34 (21)
		Female	127 (79)
Profession	Physician		
		Consultant	26 (15)
		Registrar/House Officer	45 (28)
		Medical Officer	26 (16)
	Non-physician		
		Nurse/Nurse-midwife	62 (39)
		Community Health Extension Worker/Officer	9 (6)

For peer review only - http://bmjopen.bmj.com/site/about/guidelines.xhtml

Years of practice	< 5 years	28 (17)
	5 - 10 years	62 (37)
	11 - 15 years	35 (21)
	16 - 20 years	20 (12)
	> 20 years	21 (13)
Location of practice	North East	2 (1)
	North West	7(4)
	North Central*	100 (60)
	South East	12 (7)
	South West	32(19)
	South South	14 (8)
Type of healthcare facility	Government - Tertiary care	72 (43)
	Government - Secondary care	34 (20)
	Government - Primary care	20 (12)
	Private	41 (25)
Specialty	General Paediatrics	98 (64)
	Subspecialty Paediatrics	22 (14)
	Other specialties	34 (22)

*North Central: Abuja Federal Capital Territory (FCT), the capital city of Nigeria, is located in North Central Nigeria and was the location of the conference.

Type and location of practice

Respondents were mostly in general practice (98, 64%) with fewer in subspecialty paediatrics (22, 14%). Most respondents had practiced for 10 years or less (90, 54%) and many practiced in a tertiary care setting (72, 43%). The majority of respondents practice in the North Central (100, 60%) or South West parts of Nigeria (32, 19%).

Access to simulation-based training facilities

Table 2 shows the distribution of respondents with SBT facilities at their facility by profession, years in practice, type and location of practice. There were no differences in access to SBT. Comparatively fewer respondents reported having a skills-based simulation lab at their centre (22, 23% physicians vs. 21, 34% nurses, p=0.120).

Table 2. Access to simulation-based training in health facilities

For peer review only

For peer review only - http://bmjopen.bmj.com/site/about/guidelines.xhtml

Respondent characteristics N=155	Facilities available for simulation-based training n (%)	P-value
**Profession**		NS
Physician	62 (66)	
Nurse	37 (61)	
**Years in practice**		NS
> 10 years	44 (62)	
≤ 10 years	54 (66)	
**Type of facility**		NS
Government	70 (61)	
Private	28 (70)	
**Geographic location of practice**		NS
North	59 (61)	
South	39 (68)	

North = North-East, North-Central, North-West Nigeria geopolitical zones
South = South-West, South-East, South-South Nigeria geopolitical zones

Exposure to simulation-based training

Where facilities were available for SBT, most physicians and nurses reported the use of simulation facilities for teaching (physicians 62, 65%; nurses 34, 55%). There was low reported use for research (physicians 6, 6%; nurses 10, 16%) and examination purposes (physicians 21, 22%; nurses 6, 10%). Manikin-based training was more frequently reported than online simulation. The most reported type of training was Basic Life Support (physicians 36, 38%; nurses 18, 29%). Exposure to manikin-based training varied by type of facility and geographic location (Table 3).

Physicians were the group most likely to have been exposed to manikin-based paediatric training programs such as Helping Babies Breathe (29, 30% physicians vs. 12, 19% nurses vs. 1, 11% community health workers, $p<0.001$) or online training in neonatal resuscitation using the NRP eSIM (7, 7% physician vs. 3, 5% nurses vs. 0, 0% community healthcare workers $p<0.05$). Although the majority of physicians (91, 96%) and nurses (41, 72%) owned smartphones, and many were aware that VR simulations could be run on their personal phone (43, 47% physician vs. 28, 51% nurses), only 3% (n=5) of all respondents had experienced a VR simulation.

Table 3. Exposure to manikin-based training in Basic Life Support varies by Type and Location of Facility

Basic Life Support Manikin-based training N=158	n (%)	P-value
**Profession**		NS

For peer review only - http://bmjopen.bmj.com/site/about/guidelines.xhtml

Physician (Consultant or registrar)	36 (38)	
Nurse/nurse-midwife	18 (29)	
**Years in practice**		NS
> 10 years	24 (32)	
< 10 years	30 (33)	
**Type of facility**		<0.001
Government	30 (36)	
Private	23 (58)	
**Geographic location**		<0.01
North	25 (25)	
South	28 (48)	

North = North-East, North-Central, North-West Nigeria geopolitical zones
South = South-West, South-East, South-South Nigeria geopolitical zones

Challenges to simulation-based training

Respondents identified challenges to having a skills-based simulation lab and to online (computer-based or virtual reality) simulation. Lack of curriculum and lack of funding were perceived as less of a barrier to establishing a skills-based simulation lab by respondents from private healthcare facilities compared with respondents from government facilities ($p<0.05$) (Figure 1).

Figure 1. Challenges to establishing skills-based simulation labs in private and government health facilities

Lack of awareness was the most reported challenge to using online simulation (82, 51%). Other perceived challenges to online simulation were lack of VR equipment (37, 23%) and lack of standardized VR training modules (35, 22%). Fewer respondents reported lack of internet access (24, 15%) or inconsistent power supply (21, 13%) as a challenge to online training.

Perceptions of simulation-based training

Respondents identified the advantages of SBT to include skills acquisition, provides feedback, step down training, monitoring and evaluation, debriefing/reflection, hands-on skills practice, teamwork/communication training, skills maintenance/retention, and examination purposes when patients are unavailable.

Perceptions on the value of SBT differed by experience. Healthcare workers with less experience were more likely to identify skills acquisition as an advantage of SBT (45, 59%, > 10 years vs. 64, 71% ≤ 10 years, $p<0.05$). Healthcare workers with less than or equal to 10 years of experience were more likely to identify examination purposes when patients are unavailable (23, 30% > 10 years vs. 40, 44% ≤ 10 years, $p<0.05$), while those with more than 10 years of

For peer review only

For peer review only - http://bmjopen.bmj.com/site/about/guidelines.xhtml

experience identified debriefing/reflection (25, 33% > 10 years vs. 17, 19% ≤10 years) as advantages of SBT. The perceived advantages of simulation also varied significantly by the profession of respondents. See Table 4.

Table 4. Perceived advantages of simulation-based training vary by profession

Advantages of simulation-based training	Physician n (%)	Nurse n (%)	p-value
Skills acquisition	83 (86)	27 (44)	<0.001
Provides feedback	47 (49)	11 (18)	<0.001
Step down training	48 (50)	21 (34)	NS
Monitoring and evaluation	47 (49)	16 (26)	<0.01
Debriefing/reflection	34 (35)	8 (13)	<0.01
Hands-on skills practice	64 (67)	18 (29)	<0.001
Teamwork/communication training	55 (57)	23 (37)	<0.05
Skills maintenance/retention	54 (56)	15 (24)	<0.001
Examination purposes when patients are unavailable	56 (58)	9 (15)	<0.001

All respondents thought that SBT could be expanded beyond the current scope. Physicians were more likely to advocate for expanded use of simulation for continued practice after initial training (84, 88% physician vs. 39, 63% nurses, p<0.001). They were also more likely to advocate for simulation for teaching (73, 76% physicians vs. 16, 26% nurses, p<0.001), and research (65, 68% physicians vs. 14, 23% nurses, p<0.001). If facilities were available, nearly all respondents (92, 98% physicians; 52, 96% nurses) would recommend the use of online simulation for their centre.

DISCUSSION

Using data from a national survey of paediatric healthcare workers, we explored the access to and perceived utility of various simulation modalities in a resource-scarce setting. Our study found that many healthcare workers lack access to skills-based simulation labs for manikin-based training. The perceived challenges to establishing skills-based simulation labs were comparatively greater for respondents at government healthcare facilities with the greatest identified barriers being the lack of funding and access to equipment such as manikins. This is

For peer review only - http://bmjopen.bmj.com/site/about/guidelines.xhtml

in contrast with the abundance of dedicated simulation facilities in high income countries[22-24]. Dedicated spaces and equipment for SBT are only the first step, there is also a need to develop locally relevant simulation cases and to train simulation instructors in the techniques of simulation facilitation and debriefing[24,25].

The perceived utility of SBT may vary by profession and setting. While many of our respondents identified specific ways in which SBT could be used, their responses varied by profession and experience. A variety of approaches have been described for interprofessional education including role play, manikin-based and virtual simulations. Interprofessional curricula may have differing impacts on learners of different professions[26-28]. Interprofessional virtual simulations have been shown to lead to varying changes in attitudes in for students of different health professions[28]. It is therefore reasonable to infer that healthcare workers in different professions may benefit from SBT in different ways.

Healthcare workers were open to the expansion of simulation for teaching, continuing education and research and supported the introduction of online SBT. Online SBT is made more feasible than manikin-based simulation in resource-scarce settings by the widespread availability of mobile phones[29]. We confirmed a high percentage of smartphone use among healthcare workers in our study and low concern for potential barriers such as lack of internet access or inconsistent power supply. The integration of SBT into medical and nursing school curricula provides early exposure to SBT[24]. Establishing simulation programs at public and private healthcare facilities would enable the development of contextually appropriate simulation curricula and instructor courses in simulation facilitation, debriefing and research[22,23].

A broad grass-roots approach that engages stakeholders in training institutions, state and national ministries of health, ministries of education, industry and health professional organizations is needed to support the integration of SBT into pre-service training and continuing education programs for in-service healthcare workers. Continuing education programs support the acquisition and retention of skills after initial training and have been important sources of sustainable funding for SBT in high income settings[7,13-16]. These mechanisms may be leveraged to support SBT in resource-scarce settings.

This study had some limitations. This was a cross-sectional survey developed by the authors and was not a validated instrument.The data were obtained by self-report and could be subject to selection and recall bias. The survey was administered to attendees at a national paediatric conference. Although respondents worked at both training and non-training institutions and in both public and private settings, their attendance at the conference may indicate that they may be more likely to be supportive of academic pursuits, including SBT. While physicians (both consultants and registrars) and nurses were represented in this study, other cadres of healthcare workers including community health extension workers and medical officers were not well-represented and the utilization of simulation in these groups could be a subject for future study.

CONCLUSION

For peer review only - http://bmjopen.bmj.com/site/about/guidelines.xhtml
For peer review only

The access of healthcare workers to SBT is limited in resource-scarce settings. While acknowledging the challenges of lack of awareness, limited access to equipment and funding, respondents identified many areas in which SBT has utility including skills acquisition, hands-on skills practice and communication training. Healthcare workers were open to the use of online SBT and expressed the need to expand SBT beyond the current scope for pre-service and in-service health professional training in Nigeria.

ACKNOWLEDGEMENTS

We would like to acknowledge the healthcare workers who participated in this study.

FUNDING

This work was supported by the Bill and Melinda Gates Foundation, grant number OPP1169873.

AUTHOR CONTRIBUTIONS

All authors have made substantial contributions to the planning, conduct, analyses and interpretation of findings and reporting of the work described in the article and have agreed to be accountable for all aspects of the work, its accuracy and integrity. RU is responsible for the overall content as guarantor. RU and CE formulated the study objectives and survey. IF, BE and PA assisted CE with data collection. EC assisted with data entry. CS performed statistical analysis. RU wrote the first draft of the manuscript and revised and amended it with input from all authors who also approved the final version to be published. RU is the corresponding author. The corresponding author attests that all listed authors meet authorship criteria and that no others meeting the criteria have been omitted.

COMPETING INTEREST: None declared

PATIENT CONSENT FOR PUBLICATION: Not required.

DATA AVAILABILITY STATEMENT: All data relevant to the study are included in the article.

REFERENCES

1. Sawyer T, Sierocka-Castaneda A, Chan D, Berg B, Lustik M, Thompson M. Deliberate practice using simulation improves neonatal resuscitation performance. *Simulation in Healthcare.* 2011;6(6):327-336.
2. Sawyer T, Gray MM. Procedural training and assessment of competency utilizing simulation. *Semin Perinatol.* 2016;40(7):438-446.
3. Kolb DA. Learning styles and disciplinary differences. *The modern American college.* 1981:232-255.
4. Kolb AY, Kolb DA. Learning styles and learning spaces: Enhancing experiential learning in higher education. *Academy of management learning & education.* 2005;4(2):193-212.
5. Ericsson KA. Deliberate practice and the acquisition and maintenance of expert performance in medicine and related domains. *Academic medicine.* 2004;79(10):S70-S81.

For peer review only - http://bmjopen.bmj.com/site/about/guidelines.xhtml

6. Smidt A, Balandin S, Sigafoos J, Reed VA. The Kirkpatrick model: A useful tool for evaluating training outcomes. *http://dxdoiorg/101080/13668250903093125.* 2009.
7. Sawyer T, Umoren RA, Gray MM. Neonatal resuscitation: advances in training and practice. *Adv Med Educ Pract.* 2017;8:11-19.
8. Ersdal HL, Singhal N, Msemo G, et al. Successful implementation of Helping Babies Survive and Helping Mothers Survive programs—An Utstein formula for newborn and maternal survival. *PloS one.* 2017;12(6):e0178073.
9. Wetzel EA, Lang TR, Pendergrass TL, Taylor RG, Geis GL. Identification of latent safety threats using high-fidelity simulation-based training with multidisciplinary neonatology teams. *Joint Commission Journal on Quality and Patient Safety.* 2013;39(6):268-268.
10. Patterson MD, Geis GL, Falcone RA, LeMaster T, Wears RL. In situ simulation: detection of safety threats and teamwork training in a high risk emergency department. *BMJ quality & safety.* 2013;22(6):468-477.
11. Pearlman SA, Zern SC, Blackson T, Ciarlo JA, Mackley AB, Locke RG. Use of neonatal simulation models to assess competency in bag-mask ventilation. *J Perinatol.* 2016;36(3):242-246.
12. Hawkes GA, Malik Y, Livingstone V, Dempsey EM, Ryan CA. End user bag-mask ability and perceptions of two infant resuscitation mannequins. *Acta Paediatr.* 2016;105(3):281-285.
13. Bang A, Patel A, Bellad R, et al. Helping Babies Breathe (HBB) training: What happens to knowledge and skills over time? *BMC Pregnancy and Childbirth.* 2016;16(1):364.
14. Singhal N, Lockyer J, Fidler H, et al. Helping Babies Breathe: global neonatal resuscitation program development and formative educational evaluation. *Resuscitation.* 2012;83(1):90-96.
15. Aggarwal R, Mytton OT, Derbrew M, et al. Training and simulation for patient safety. *Quality and Safety in Health Care.* 2010;19(Suppl 2):i34.
16. Curtis MT, DiazGranados D, Feldman M. Judicious Use of Simulation Technology in Continuing Medical Education. *Journal of Continuing Education in the Health Professions.* 2012;32(4):255-260.
17. Spooner N, Hurst S, Khadra M. Medical simulation technology: Educational overview, industry leaders, and what's missing. *Hospital topics.* 2012;90(3):57-64.
18. Umoren RA PJ, Sweigart L, Rybas N, Gossett E, Johnson M, Allen M, Scott PJ, Truman B, Das RR. TeamSTEPPS Virtual Teams: Interactive Virtual Team Training and Practice for Health Professional Learners. *Creative Nursing.* 2017;23(3).
19. Umoren R, Stadler DJ, Gasior SL, Al-Sheikhly D, Truman B, Lowe C. Global collaboration and team-building through 3D virtual environments. *Innovations in Global Medical and Health Education.* 2014:1.
20. O'Lynn CE. Gender-based barriers for male students in Nursing education programs: Prevalence and perceived importance. *Journal of Nursing Education.* 2004;43(5):229-236.
21. Vassar L. How medical specialties vary by gender. *AMA Wire.* 2015;1.
22. Ziv A, Erez D, Munz Y, et al. The Israel Center for Medical Simulation: a paradigm for cultural change in medical education. *Acad Med.* 2006;81(12):1091-1097.
23. Akaike M, Fukutomi M, Nagamune M, et al. Simulation-based medical education in clinical skills laboratory. *J Med Invest.* 2012;59(1-2):28-35.
24. Lopreiato JO, Sawyer T. Simulation-based medical education in Pediatrics. *Acad Pediatr.* 2015;15(2):134-142.
25. Sawyer T, Eppich W, Brett-Fleegler M, Grant V, Cheng A. More Than One Way to Debrief. *Simul Healthc.* 2016;11(3):209-217.
26. Carpenter J, Dickinson H. *Interprofessional education and training.* policy press; 2016.

For peer review only - http://bmjopen.bmj.com/site/about/guidelines.xhtml

27. West C, Graham L, Palmer RT, et al. Implementation of interprofessional education (IPE) in 16 U.S. medical schools: Common practices, barriers and facilitators. *J Interprof Educ Pract.* 2016;4:41-49.

28. Umoren RA, Scott PJ, Sweigart L, et al. A comparison of teamwork attitude changes with virtual TeamSTEPPS® simulations in health professional students. *Journal of Interprofessional Education & Practice.* 2018;10:51-55.

29. Asangansi I, Braa K. The emergence of mobile-supported national health information systems in developing countries. *Stud Health Technol Inform.* 2010;160(Pt 1):540-544.

Figure Legend

Figure 1. Challenges to establishing skills-based simulation labs in private and government health facilities

Legend: *p<0.05

For peer review only

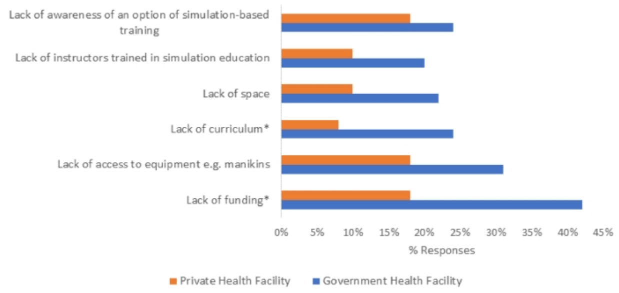

Figure 1. Challenges to establishing skills-based simulation labs in private and government health facilities
Legend: *p<0.05

338x190mm (300 x 300 DPI)

Survey on simulation based learning/practice

Please answer the following questions:

1. **What is your age range?**
   a. <21 **b.** 21-30 **c.** 31-40 **d.** 41-50 **e.** >50
2. **Sex**
   a. Male b. Female
3. **Profession**
   a. Consultant physician **b.** Senior resident/registrar **c.** Resident/registrar **d.** Nurse **e.** Other __________
4. **Years of practice**
   a. < 5 years **b.** 5-10 years **c.** 11-15 years **d.** 16-20 years
   e. >20 years
5. **Current location of practice**
   ____
6. **Type of practice**
   a. Government facility
      i. Tertiary care
      ii. Secondary care
      iii. Primary care
   b. Private practice
   c. Faith-based facility
7. **Specialty**
   a. General Pediatrics
   b. Subspecialty Pediatrics (Please specify ____________________)
   c. Other specialty (Please specify ____________________)
8. **Are you aware of the use of simulation-based training at your institution?**
   a. Yes **b.** No
9. **If yes in Q8, in what capacity does your institution use simulation-based training? (select all that apply)**
   a. Teaching **b.** Research **c.** Examination
10. **Does your center have a skills-based simulation lab?**
    a. Yes **b.** No
11. **If yes in Q10, what is the skills-based simulation lab available for? (select all that apply)**
    a. Skills practice e.g. HBB Newborn corner **b.** Teaching **c.** Research **d.** Examination
12. **If no in Q10, what are the challenges to having a skills-based simulation lab at your center?**
    a. Lack of funding

For peer review only

- **b.** Lack of access to equipment e.g. manikins
- **c.** Lack of curriculum
- **d.** Lack of space
- **e.** Lack of instructors trained in simulation education
- **f.** Lack of awareness of an option for simulation-based training

13. Which modality of simulation based training have you been exposed to? (select all that apply)

- **a.** Manikin-based training
  - i. HBB
  - ii. NRT
  - iii. PALS
  - iv. ENCC
  - v. BLS
- **b.** Online (computer-based) simulation
  - i. NRP eSIM™
  - ii. HeartCode™ (PALS online course)
  - iii. Online Basic Life Support course
  - iv. Online ACLS course

14. Are you aware of virtual reality simulation training?

a. Yes **b.** No

15. If yes in Q14, when or where were you exposed to virtual reality simulation?

---

16. If no in Q14, what are the challenges to online (computer-based or virtual reality) simulation?

- **a.** Lack of internet access
- **b.** Lack of standardized VR training modules
- **c.** Inconsistent power supply
- **d.** Lack of access to VR equipment and computers

17. Which of these advantages of simulation-based training are you aware of (select all that apply)?

- **a.** Skills acquisition
- **b.** Provides feedback
- **c.** Step down training
- **d.** Monitoring and evaluation
- **e.** Debriefing/reflection
- **f.** Hands-on skills practice
- **g.** Teamwork/communication training
- **h.** Skills maintenance/retention
- **i.** Examination purposes when patients are unavailable

18. What type of mobile phone device do you own or use? (Choose all that apply)

- **a.** Tablet (Eg. Ipad, tablets)

For peer review only - http://bmjopen.bmj.com/site/about/guidelines.xhtml
For peer review only

- **b.** Smart Phone (eg. Iphone, Samsung, Techno, Nexus, Infinix etc.)
- **c.** Feature phone (Eg. Does some gprs based activities)
- **d.** Basic (Eg. Used for call and SMS only)

19. What is the manufacturer and model of your phone/mobile device?

____

20. If you are using an android enabled device, what android version does your device run (To find out, Goto |Settings->General->AboutDevice| and look for version number)

- **a.** Gingerbread (version 2.3)
- **b.** Ice Cream Sandwich (version 4.0)
- **c.** Jelly Bean (version 4.1 – 4.3)
- **d.** KitKat (Version 4.4)
- **e.** Lollipop (Version 5.0 – 5.1)
- **f.** Marshmallow (Version 6.0)
- **g.** Nougat (Version 7)
- **h.** Oreo (Version 8)

21. Do you use mobile device currently for your work?

a. Yes **b.** No

22. Do you think simulation based training could be expanded beyond the current scope?

a. Yes **b.** No

23. If yes in Q22, in what way should simulation-based training be expanded in Nigeria?

- **a.** Continued practice after initial training
- **b.** Teaching
- **c.** Research

24. If all facilities were available, would you recommend online simulation for your center?

a. Yes b. No

25. If No in Q24, please state your reason(s)____

For peer review only - http://bmjopen.bmj.com/site/about/guidelines.xhtml

Reporting checklist for cross sectional study.

Based on the STROBE cross sectional guidelines.

Instructions to authors

Complete this checklist by entering the page numbers from your manuscript where readers will find each of the items listed below.

Your article may not currently address all the items on the checklist. Please modify your text to include the missing information. If you are certain that an item does not apply, please write "n/a" and provide a short explanation.

Upload your completed checklist as an extra file when you submit to a journal.

In your methods section, say that you used the STROBE cross sectionalreporting guidelines, and cite them as:

von Elm E, Altman DG, Egger M, Pocock SJ, Gotzsche PC, Vandenbroucke JP. The Strengthening the Reporting of Observational Studies in Epidemiology (STROBE) Statement: guidelines for reporting observational studies.

		Reporting Item	Page Number
**Title and abstract**			
Title	#1a	Indicate the study's design with a commonly used term in the title or the abstract	1

For peer review only

Abstract	#1b	Provide in the abstract an informative and balanced summary of what was done and what was found	1
**Introduction**			
Background / rationale	#2	Explain the scientific background and rationale for the investigation being reported	2
Objectives	#3	State specific objectives, including any prespecified hypotheses	2
**Methods**			
Study design	#4	Present key elements of study design early in the paper	2
Setting	#5	Describe the setting, locations, and relevant dates, including periods of recruitment, exposure, follow-up, and data collection	2
Eligibility criteria	#6a	Give the eligibility criteria, and the sources and methods of selection of participants.	3
	#7	Clearly define all outcomes, exposures, predictors, potential confounders, and effect modifiers. Give diagnostic criteria, if applicable	n/a
Data sources / measurement	#8	For each variable of interest give sources of data and details of methods of assessment (measurement). Describe comparability of assessment methods if there is more than one group. Give information separately for for exposed and unexposed groups if applicable.	3

For peer review only

Bias	#9	Describe any efforts to address potential sources of bias	3
Study size	#10	Explain how the study size was arrived at	n/a
Quantitative variables	#11	Explain how quantitative variables were handled in the analyses. If applicable, describe which groupings were chosen, and why	4
Statistical methods	#12a	Describe all statistical methods, including those used to control for confounding	4
Statistical methods	#12b	Describe any methods used to examine subgroups and interactions	4
Statistical methods	#12c	Explain how missing data were addressed	4
Statistical methods	#12d	If applicable, describe analytical methods taking account of sampling strategy	4
Statistical methods	#12e	Describe any sensitivity analyses	4
**Results**			
Participants	#13a	Report numbers of individuals at each stage of study—eg numbers potentially eligible, examined for eligibility, confirmed eligible, included in the study, completing follow-up, and analysed. Give information separately for for exposed and unexposed groups if applicable.	4
Participants	#13b	Give reasons for non-participation at each stage	n/a

For peer review only

Participants	#13c	Consider use of a flow diagram	n/a
Descriptive data	#14a	Give characteristics of study participants (eg demographic, clinical, social) and information on exposures and potential confounders. Give information separately for exposed and unexposed groups if applicable.	4
Descriptive data	#14b	Indicate number of participants with missing data for each variable of interest	Tables 1-4
Outcome data	#15	Report numbers of outcome events or summary measures. Give information separately for exposed and unexposed groups if applicable.	n/a
Main results	#16a	Give unadjusted estimates and, if applicable, confounder-adjusted estimates and their precision (eg, 95% confidence interval). Make clear which confounders were adjusted for and why they were included	4
Main results	#16b	Report category boundaries when continuous variables were categorized	4
Main results	#16c	If relevant, consider translating estimates of relative risk into absolute risk for a meaningful time period	4
Other analyses	#17	Report other analyses done—e.g., analyses of subgroups and interactions, and sensitivity analyses	n/a
**Discussion**			
Key results	#18	Summarise key results with reference to study objectives	6

For peer review only

Limitations	#19	Discuss limitations of the study, taking into account sources of potential bias or imprecision. Discuss both direction and magnitude of any potential bias.	7
Interpretation	#20	Give a cautious overall interpretation considering objectives, limitations, multiplicity of analyses, results from similar studies, and other relevant evidence.	7
Generalisability	#21	Discuss the generalisability (external validity) of the study results	7
**Other Information**			
Funding	#22	Give the source of funding and the role of the funders for the present study and, if applicable, for the original study on which the present article is based	1

The STROBE checklist is distributed under the terms of the Creative Commons Attribution License CC-BY. This checklist was completed on 31. August 2019 using https://www.goodreports.org/, a tool made by the EQUATOR Network in collaboration with Penelope.ai

For peer review only
